# BAR502/fibrate conjugates: synthesis, biological evaluation and metabolic profile

**DOI:** 10.3389/fchem.2024.1425867

**Published:** 2024-07-17

**Authors:** Claudia Finamore, Simona De Marino, Chiara Cassiano, Giuliano Napolitano, Pasquale Rapacciuolo, Silvia Marchianò, Michele Biagioli, Rosalinda Roselli, Cristina Di Giorgio, Carmen Festa, Stefano Fiorucci, Angela Zampella

**Affiliations:** ^1^ Department of Pharmacy, University of Naples, Naples, Italy; ^2^ Department of Medicine and Surgery, University of Perugia, Perugia, Italy

**Keywords:** hybrid prodrug, BAR502, fibrates, bile acid receptors, metabolic profile, stability, cell permeation, anti-inflammatory activity

## Abstract

BAR502, a bile acid analogue, is active as dual FXR/GPBAR1 agonist and represents a promising lead for the treatment of cholestasis and NASH. In this paper we report the synthesis and the biological evaluation of a library of hybrid compounds prepared by combining, through high-yield condensation reaction, some fibrates with BAR502.The activity of the new conjugates was evaluated towards FXR, GPBAR1 and PPARα receptors, employing transactivation or cofactor recruitment assays. Compound 1 resulted as the most promising of the series and was subjected to further pharmacological investigation, together with stability evaluation and cell permeation assessment. We have proved by LCMS analysis that compound 1 is hydrolyzed in mice releasing clofibric acid and BAR505, the oxidized metabolite of BAR502, endowed with retained dual FXR/GPBAR1 activity.

## Introduction

In the context of liver diseases, nonalcoholic fatty liver disease (NAFLD) and non-alcoholic steatohepatitis (NASH)- the liver counterpart of the metabolic syndrome-represent two highly prevalent human disorders due to excessive deposition of lipids in liver leading to metabolically stressed hepatocytes with activation of cell death and proinflammatory signaling pathways. Despite the epidemic proportion with about 20%–30% of Western and Asian population affected by NAFLD/NASH, and the large number of small molecules with different mechanisms of action currently under investigation, nowadays there is no FDA or European Medicines Agency (EMA) approved therapy for NASH.

From a physio-pathological point of view, NASH is a complex disease involving several targets and pathways and is today well-accepted that treating NASH requires multi-targeted approaches, with combination of molecules acting on different targets or with multi-targeting molecules.

With their broad expression in liver compartments, metabolic nuclear receptors are considered suitable targets in the discovery of novel therapeutics for NAFLD/NASH.

As ligand-activated transcription factors, nuclear receptors (NRs) regulate the expression of genes responsible for physiological and pathological processes including reproduction, metabolism of xeno- and endobiotic, inflammation, cell proliferation and fibrosis. Among the metabolic NRs, farnesoid X receptor (FXR), broadly expressed in all relevant liver cellular compartments, has become a central therapeutic target for cholestatic and fatty liver diseases. FXR is the master gene that orchestrates bile acids (BAs) homeostasis (Makishima et al., 1999; Parks et al., 1999; Wang et al., 1999), regulating their synthesis, uptake, and secretion (Goodwin et al., 2000). As consequence of abnormal levels in bile acid concentrations that associate with liver fibrosis and inflammation (Fiorucci and Baldelli, 2009; Fiorucci et al., 2010; Fiorucci et al., 2014; Sepe et al., 2015), FXR has been identified as an appealing target for the treatment of primary biliary cirrhosis (PBC), NAFLD, and NASH (Fiorucci and Baldelli, 2009; Fiorucci et al., 2011; Fiorucci et al., 2012a; Fiorucci et al., 2012b).

FXR plays also a crucial beneficial role in triglyceride and cholesterol homeostasis, as well as in glucose metabolism (Fiorucci et al., 2004; Cariou et al., 2006; Zhang et al., 2006; Fiorucci et al., 2007; Fiorucci et al., 2009; Cipriani et al., 2010; Mencarelli et al., 2013; Swanson et al., 2013).

In addition to FXR and other nuclear hormone receptors, BAs can also signal through a membrane-receptor (GPBAR1/TGR5/M-BAR) (Maruyama et al., 2002; Kawamata et al., 2003; Fiorucci and Distrutti, 2015; Copple and Li, 2016), and responses to GPBAR1 activation include increased energy expenditure, improved intestinal motility, glucose metabolism and insulin sensitivity (Watanabe et al., 2006; Thomas et al., 2009). The latter two occur through the release of the glucagon-like peptide 1 (GLP-1) by intestinal L cells upon GPBAR1 activation (Parker et al., 2012).

Among endogenous BAs family, the activity toward FXR and GPBAR1 is structure dependent, with chenodeoxycholic acid (CDCA), and its conjugated forms, the most potent endogenous FXR activators and lithocholic acid (LCA) and its tauro-conjugated (TLCA) the strongest natural agonists of GPBAR1.

Starting from the structure of BAs and considering the convergence of both receptors in regulating several aspects of metabolic disorders, many synthetic ligands endowed with dual or selective activity toward GPBAR1 and FXR have been designed and developed for the treatment of NASH (Tiwari and Maiti, 2009; Fiorucci, et al., 2020; Ratziu et al., 2022).

Among these modulators, several potent and selective steroidal and non-steroidal FXR agonists have completed phase II/phase III trials in NASH patients. While various degrees of efficacy in improving histopathology features of NASH have been reported, none of these agents has been reported to completely reverse NASH features, and side effects have emerged (Tiwari and Maiti, 2009), suggesting that combination therapies might be required to im-prove efficacy and reduce side effects (Dufour et al., 2020; Trauner and Fuchs, 2022).

As consequence of the limited success of NASH-monotherapy, it is well-accepted that future treatment will require combination therapy in most patients, and several combination trials are now underway (Dufour et al., 2020; Powell et al., 2021). Currently, drug classes suitable for combination, acting with different mechanisms of action targeting hepatic steatosis, inflammation and fibrosis, are FXR agonists, PPARs agonists, thyroid hormone receptor beta agonists, carriers inhibitors (mitochondria pyruvate carrier, sodium/glucose transport protein 2), metabolic enzyme inhibitors [stearoyl-CoA desaturase-1 (SCD-1), acetyl-CoA carboxylase (ACC), hepatic fructokinase], fibroblast growth factor 21 (FGF21) agonists, glucagon-like peptide-1 (GLP-1) receptor agonists, and chemokine receptor (CCR) inhibitors (Dufour et al., 2020).

PPARs are a group of NRs that fine-tune lipid and glucose metabolism and regulate inflammation and fibrosis (Pawlak et al., 2015; Dubois et al., 2017); besides fibrates, PPARα ligands, show anti-steatogenic activity in animal models of NAFLD and inhibit adipocyte hypertrophy and insulin resistance inducing fatty acid β-oxidation in adipose tissue (Monk and Todd, 1987; Schoonjans et al., 1996; Fruchart et al., 1998; Jeong and Yoon, 2009), small pilot NASH studies did not show convincing efficacy (Laurin et al., 1996).

However, several studies have demonstrated beneficial effects of fibrates in cholestasis and encouraging first results have also been reported in primary sclerosing cholangitis (PSC) in combination with ursodeoxycholic acid (UDCA) (Lemoinne et al., 2018). UDCA is the first-line treatment in PBC patients, reducing disease progression and increasing survival rate and quality of life (Harms et al., 2019). In addition, UDCA exerts some beneficial effects in animal model of NASH, but it failed to improve liver histopathology and did not reduce hepatocytes ballooning and liver fibrosis. The above preclinical data is also confirmed in clinical trials reporting UDCA as only partially effective in reversing histopathology and biochemical features of NASH as a single drug-treatment (Lindor et al., 2004; Leuschner et al., 2010; Ratziu, 2012; Marchianò et al., 2022).

Over the last 10 years, our research group harnessed bile acid chemical scaffold in the identification of a new armamentarium in NAFLD and NASH therapy (Festa et al., 2014; Finamore et al., 2016; Sepe et al., 2016). Among all molecules discovered, **BAR502**, a dual FXR and GPBAR1 agonist, effectively reduced steatosis and fibrosis in a rodents’ model and is currently advancing to clinical stage (Carino et al., 2017; Carino et al., 2019).

Indeed, **BAR502** did not completely reverse liver damage and exerted no effects on lipid protein profile in mice fed a high fat, high cholesterol diet but the administration in a mouse model of NAFLD/NASH in combination with UDCA exerted beneficial effects re-versing almost completely the liver features of NASH (Marchianò et al., 2023).

Continuing along this route, in this paper we propose a new series of hybrid compounds obtained by the condensation of **BAR502** with fibrates, varying the conjugation position (C-23 and/or C-3) to develop new leads for the treatment of metabolic disorders. The design of hybrid drugs, in which two or more pharmacologically active entities are covalently joined in the same molecular skeleton, is usually performed in medicinal chemistry with the aim to obtain a single synergistic “super molecule”, that retains the pharmacological actions of each counterpart or is endowed with higher effect than the sum of each individual parts. The development of this type of compounds represents an interesting approach to discover new therapeutic strategies and different bile acid-based conjugated containing natural molecules have been explored in cancer therapy to enhance the biological activity or to reduce multidrug resistance (Navacchia et al., 2021).

**FIGURE 1:**
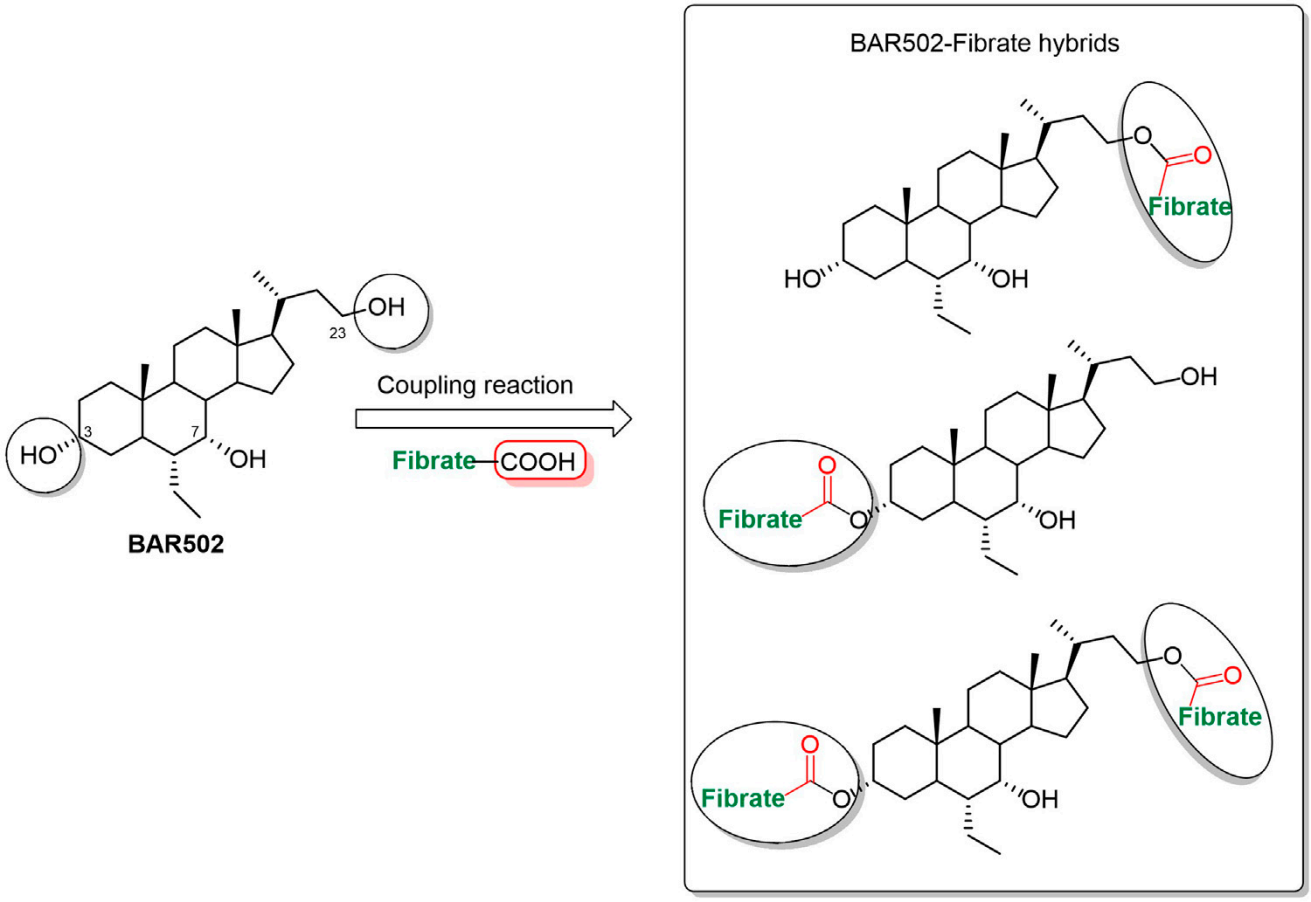
BA-fibrate hybrids sketch.

A panel of BAR502-fibrate conjugates (**1**–**9**) was prepared exploiting the hydroxyl groups on **BAR502** and the carboxylic moiety of fibrates as conjugation points through a condensation reaction (Figure 1). The ester linkage is the common cleavable chemical bond used in the synthesis of conjugated compounds that act as prodrugs, providing, under enzymatic or physiological conditions, the release of the two active molecular entities to strengthen the pharmacological effect.

The activity of BAR502-fibrate conjugates (**1**–**9**) was evaluated *in vitro* on FXR, GPBAR1 and PPARα and the most promising candidate, compound **1**, was subjected to further pharmacological evaluation.

Moreover, we performed *in vitro* and *in vivo* studies to elucidate the metabolic profile, stability and cell permeation of hybrid compound **1** reporting also the synthesis of its major metabolite.

## Materials and methods

### Chemical synthesis

#### General remarks

NMR spectra were acquired on a Bruker Avance NEO 400 and 700 spectrometers with a RT-DR-BF/1H-5 mm-OZ SmartProbe (^1^H at 400 and 700 MHz and ^13^C at 100 and 175 MHz respectively) and recorded in CD_3_OD (δ_H_ 3.30 and δ_C_ 49.0 ppm) and CDCl_3_ (δ_H_ 7.26 and δ_C_ 77.0 ppm). Detected signals were in accordance with the proposed structures. Chemical shifts (δ) are reported in ppm and referenced to the residual undeuterated solvent; coupling constant (*J*) values are given in Hertz (Hz). Spin multiplicities are given as s (singlet), br s (broad singlet), d (doublet), t (triplet), or m (multiplet). High-resolution electrospray ionization mass spectrometry (ESI-MS) spectra were performed with an LTQ-XL equipped with an Ultimate 3000 HPLC system (Thermo Fisher Scientific) mass spectrometer. HPLC was performed with a Waters Model 510 pump equipped with Waters^®^ Rheodine injector and a differential refractometer, model 401. Reaction progress was monitored via thin layer chromatography (TLC) on Alugram silica gel G/UV254 plates.

Silica gel MN Kiesel gel 60 (70–230 mesh) from Macherey-Nagel was used for flash chromatography. Silica gel (200–400 mesh) from Macherey-Nagel Company was used for flash chromatography. **BAR502** was supplied by BAR Pharmaceuticals s.r.l.

Chemicals and solvents were supplied from Merk-Sigma or Zentek, Inc. with the following exceptions. Hexane, ethyl acetate, chloroform, dichloromethane, tetrahydrofuran, and triethylamine were distilled from calcium hydride immediately prior to use. Methanol was dried from magnesium methoxide as follows. Magnesium turnings (5 g) and iodine (0.5 g) were refluxed in a small (50–100 mL) quantity of methanol until all the magnesium has reacted. The mixture was diluted (up to 1 L) with reagent-grade methanol, refluxed for 2–3 h, and then distilled under nitrogen. All reactions were carried out under argon atmosphere using flame-dried glassware. The purity of all final compounds was determined to be greater than 95% by analytical HPLC, MS and NMR analysis.

#### General method for the coupling of BAR502-fibrate conjugate derivatives (1, 3, 4, 6, 7 and 9)

Fibrate acids (clofibric acid, fenofibric acid or gemfibrozil) were dissolved in anhydrous DMF (10 mL), and then diisopropylethylamine (8 eq) and coupling reagents EDC•HCl (4 eq) and HOBt (4 eq) were added. After 30 min, the **BAR502** were added to each reaction. The reactions were stirred at room temperature overnight. When the reactions were completed, the solvent was removed under vacuum and the crude residues were extracted with H_2_O/EtOAc (50 mL) for three times. The organic layers were dried over Na_2_SO_4_, filtered, and concentrated under reduced pressure.

#### Preparation of compounds 1 and 3

The reaction of **BAR502** (200 mg, 0.51 mmol) with clofibric acid (3 eq) furnished a mixture of compounds **1** and **3**, efficiently separated by HPLC using a reverse-phase column (Luna C18, 10 μm, 10 mm i.d. x 250 mm) and MeOH/H_2_O 95:5 as eluent (flow rate 3 mL/min) giving pure compounds **1** (70.7 mg, t_R_ = 9.7 min) and **3** (24.3 mg, t_R_ = 18.8 min).

Compound **1**. Selected ^1^H NMR (400 MHz, CDCl_3_): δ_H_ 7.20 (2H, d, *J* = 8.7 Hz, H-3″ and H-5″ of clofibric unit), 6.77 (2H, d, *J* = 8.7 Hz, H-2″ and H-6” of clofibric unit), 4.22 (1H, m, H-23α), 4.19 (1H, m, H-23β), 3.71 (1H, s, H-7β), 3.44 (1H, m, H-3β), 1.59 (6H, s, CH_3_-3′ and CH_3_-4’ of clofibric unit), 0.92 (6H, ovl, CH_3_-21 and CH_3_-25), 0.90 (3H, s, CH_3_-19), 0.60 (3H, s, CH_3_-18). ^13^C NMR (100 MHz, CDCl_3_): δc 174.3, 153.9, 129.2 (2C), 126.8, 119.8 (2C), 79.4, 72.3, 70.9, 63.7, 55.9, 50.3, 45.2, 42.6, 41.2, 39.8, 39.6, 35.4, 34.4, 33.9, 33.1, 33.0, 30.6, 28.2, 25.4, 25.1 (2C), 23.6, 23.0, 22.2, 20.7, 18.5, 11.6, 11.5. ESI-MS *m/z* 571.4 [M + H-H_2_O]^+^ and 553.3 [M + H-2H_2_O]^+^.

Compound **3**. Selected ^1^H NMR (400 MHz, CDCl_3_): δ_H_ 7.20 (4H, d, *J* = 8.7 Hz, H-3″ and H-5″ of clofibric units), 6.80–6.77 (4H, d, *J* = 8.7 Hz, H-2″ and H-6” of clofibric units), 4.62 (1H, m, H-3β), 4.21 (1H, m, H-23α), 4.19 (1H, m, H-23β), 3.71 (1H, s, H-7β), 1.59–1.57 (12H, s, CH_3_-3′ and CH_3_-4’ of clofibric units), 0.92 (6H, ovl, CH_3_-21 and CH_3_-25), 0.91 (3H, s, CH_3_-19), 0.60 (3H, s, CH_3_-18). ^13^C NMR (100 MHz, CDCl3): δc 174.1, 173.3, 154.1 (2C), 129.1 (2C), 128.9 (2C), 127.0, 126.9, 120.4 (2C), 120.1 (2C), 79.5, 79.3, 76.0, 70.5, 63.7, 55.8, 50.3, 45.0, 42.7, 41.0, 39.9, 39.4, 35.5, 35.0, 34.3, 33.0, 32.9, 29.2, 28.3, 26.4, 25.6, 25.3, 25.2, 25.1, 23.6, 23.1, 22.1, 20.7, 18.6, 11.6 (2C). ESI-MS m/z 767.4 [M + H-H_2_O]^+^.

#### Preparation of compounds 4 and 6

The product obtained from the reaction of **BAR502** (50 mg, 0.13 mmol) with fenofibric acid, using the same conditions previously described, was purified by HPLC (reverse-phase column, Luna C18, 10 μm, 10 mm i.d. x 250 mm) and MeOH/H_2_O 95:5 as eluent (flow rate 3 mL/min) giving compounds **4** (32 mg, t_R_ = 11.1 min) and **6** (4.6 mg, t_R_ = 31.2 min).

Compound **4**. Selected ^1^H NMR (400 MHz, CDCl_3_): δ_H_ 7.74 (2H, d, *J* = 8.6 Hz, H-3″ and H-5″ of fenofibric unit), 7.70 (2H, d, *J* = 8.4 Hz, H-2‴ and H-6‴ of fenofibric unit), 7.45 (2H, d, *J* = 8.4 Hz, H-3‴ and H-5‴ of fenofibric unit), 6.85 (2H, d, *J* = 8.6 Hz, H-2″ and H-6″ of fenofibric unit), 4.22 (1H, m, H-23α), 4.20 (1H, m, H-23β), 3.68 (1H, s, H-7β), 3.40 (1H, m, H-3β), 1.68 (6H, s, CH_3_-3′and CH_3_-4′ of fenofibric unit), 0.89 (6H, ovl, CH_3_-21 and CH_3_-25), 0.87 (3H, ovl, CH_3_-19), 0.58 (3H, s, CH_3_-18). ^13^C NMR (100 MHz, CDCl_3_): δ_C_ 194.0, 174.0, 159.5, 138.5, 136.5, 132.0 (2C), 131.0 (2C), 130.0, 128.5 (2C), 117.2 (2C), 79.0, 72.3, 70.8, 63.8, 55.8, 50.3, 44.9, 42.3, 41.2, 39.6, 39.3, 35.4, 34.2, 33.9, 32.9, 32.7, 30.4, 28.2, 25.4, 25.1 (2C), 23.3, 23.0, 21.8, 20.5, 18.5, 11.7, 11.4. ESI-MS m/z 675.4 [M + H-H_2_O]^+^ and 657.4 [M + H-2H_2_O]^+^.

Compound **6**. Selected ^1^H NMR (400 MHz, CDCl_3_): δ_H_ 7.73 (8H, ovl, H-3″ and H-5, H-2‴ and H-6‴ of fenofibric units), 7.45 (4H, ovl, H-3‴ and H-5‴ of fenofibric units), 6.87 (4H, ovl, H-2″ and H-6″ of fenofibric units), 4.64 (1H, m, H-3β), 4.22 (1H, m, H-23α), 4.18 (1H, m, H-23β), 3.67 (1H, s, H-7β), 1.69 (12H, s, CH_3_-3′and H-4′ of fenofibric units), 0.82 (9H, ovl, CH_3_-21, CH_3_-25 and CH_3_-19), 0.56 (3H, s, CH_3_-18). ^13^C NMR (100 MHz, CDCl_3_): δ_C_ 194.6, 194.3, 174.0, 173.3, 160.0, 159.8, 138.5 (2C), 136.6 (2C), 132.2 (2C), 132.1 (2C), 131.4 (2C), 131.3 (2C), 130.4, 130.3, 128.7 (4C), 117.5, 117,4, 79.7, 79.5, 70.6, 64.1, 56.2, 50.6, 45.3, 42.9, 41.3, 40.1, 39.6, 35.7, 35.2, 34.6, 33.3, 33.2, 29.4, 28.4, 26.6, 25.7 (4C), 25.6, 25.5, 23.8, 23.3, 22.4, 20.8, 18.7, 11.8 (2C). ESI-MS m/z 975.4 [M + H-H_2_O]^+^.

#### Preparation of compounds 7 and 9

The product obtained from the reaction of **BAR502** (50 mg, 0.13 mmol) with gemfibrozil, using the same conditions previously described, was purified by HPLC (reverse-phase column, Luna C18, 10 μm, 10 mm i.d. x 250 mm) and MeOH/H_2_O 99.5:0.5 as eluent (flow rate 3 mL/min) giving compounds **7** (38 mg, t_R_ = 11.6 min) and **9** (1.6 mg, t_R_ = 37.6 min).

Compound **7**. Selected ^1^H NMR (400 MHz, CDCl_3_): δ_H_ 7.01 (1H, d, *J* = 7.4 Hz, H-3″ of gemfibrozil unit), 6.66 (1H, d, *J* = 7.4 Hz, H-4″ of gemfibrozil unit), 6.61 (1H, s, H-6″ of gemfibrozil unit), 4.09 (2H, m, H_2_-23), 3.91 (2H, t, *J* = 5.5 Hz, H-5′ of gemfibrozil unit), 3.68 (1H, s, H-7β), 3.42 (1H, m, H-3β), 2.31 (3H, s, CH_3_-8″ of gemfibrozil unit), 2.18 (3H, s, CH_3_-7”), 1.22 (6H, s, CH_3_-6′ and CH_3_-7’ of gemfibrozil unit), 0.97 (3H, d, *J* = 6.5 Hz, CH_3_-21), 0.91 (3H, t, *J* = 7.4 Hz, CH_3_-25), 0.90 (3H, s, CH_3_-19), 0.63 (3H, s, CH_3_-18). ^13^C NMR (100 MHz, CDCl_3_): δ_C_ 178.0, 156.3, 136.3, 130.4, 123.7, 120.4, 111.6, 72.5, 70.8, 68.0, 62.5, 56.0, 50.4, 45.2, 42.8, 41.9, 41.1, 40.0, 39.5, 37.1, 35.5 (2C), 34.6, 33.9, 33.1 (2C), 30.6, 28.3, 25.2 (3C), 23.6, 23.0, 22.3, 21.4, 20.7, 18.8, 15.7, 11.5 (2C). ESI-MS m/z 607.5 [M + H-H_2_O]^+^ and 589.5 [M + H-2H_2_O]^+^.

Compound **9**. Selected ^1^H NMR (400 MHz, CDCl_3_): δ_H_ 7.00 (2H, d, *J* = 7.4 Hz, H-3″ of gemfibrozil units), 6.66 (2H, d, *J* = 7.4 Hz, H-4″ of gemfibrozil units), 6.61 (2H, s, H-6″ of gemfibrozil units), 4.53 (1H, m, H-3β), 4.11 (2H, m, H_2_-23), 3.91 (4H, m, H-5′ of gemfibrozil units), 3.69 (1H, s, H-7β), 2.30 (6H, s, CH_3_-8″ of gemfibrozil units), 2.18 (6H, s, CH_3_-7” of gemfibrozil units), 1.20 (12H, ovl, CH_3_-6′ and CH_3_-7’ of gemfibrozil units), 0.97 (3H, d, *J* = 6.5 Hz, CH_3_-21), 0.91 (6H, ovl, H_3_-19 and H_3_-25), 0.65 (3H, s, CH_3_-18). ^13^C NMR (100 MHz, CDCl_3_): δ_C_ 177.9, 177.8, 156.5 (2C), 136.1 (2C), 129.9 (2C), 123.4 (2C), 120.9 (2C), 111.2 (2C), 74.3, 70.4, 67.7 (2C), 62.3, 55.8, 50.2, 44.9, 42.6, 41.6 (2C), 40.9, 39.5, 39.1, 36.9 (2C), 35.4, 34.8, 34.4, 32.8 (2C), 29.2 (4C), 27.3, 26.5, 24.9 (4C), 24.7, 23.3, 22.8, 21.8, 21.1, 20.5, 18.3, 15.4, 11.4 (2C). ESI-MS m/z 839.6 [M + H-H_2_O]^+^.

#### Preparation of compound 2

**BAR502** (100 mg, 0.25 mmol) was dissolved in dry pyridine (3 mL) and acetic anhydride (25 μL, 0.25 mmol) was added. After 1 h, the pyridine was concentrated under vacuum. The residue was poured into cold water (10 mL) and extracted with ethyl acetate (3 × 10 mL). The combined organic phases were dried (Na_2_SO_4_) and concentrated to give a residue that was subjected to the next step without further purification affording mainly compound **10**. Selected ^1^H NMR (400 MHz, CD_3_OD): d_H_ 4.11 (2H, m, H_2_-23), 3.65 (1H, s, H-7β), 3.53 (1H, s, H-3β), 2.00 (3H, s, COCH_3_), 0.96 (3H, d, *J* = 6.5 Hz, CH_3_-21), 0.91 (6H, ovl, CH_3_-19 and CH_3_-25), 0.67 (3H, s, CH_3_-18).

Compound **10** was subjected to a coupling reaction with clofibric acid, using the same reaction condition previously described, followed by deacetylation using catalytic amount of p-TosOH in CHCl_3_/MeOH (5:3). The reaction was neutralized with aqueous saturated solution of NaHCO_3_. After evaporation of the solvent, the residue was diluted with water and extracted with DCM (3 × 20 mL). The combined extract was washed with brine, dried with Na_2_SO_4_, and evaporated to give 200 mg of a crude residue that was purified by HPLC using a reverse-phase column (Luna C18, 10 μm, 10 mm i.d. x 250 mm) and MeOH/H_2_O 95:5 as eluent (flow rate 3 mL/min) giving compound **2** (35 mg, t_R_ = 10.0 min).

Compound **2**. Selected ^1^H NMR (400 MHz, CDCl_3_): δ_H_ 7.18 (2H, d, *J* = 8.9 Hz, H-3″ and H-5″ of clofibric unit), 6.80 (2H, d, *J* = 8.7 Hz, H-2″ and H-6” of clofibric unit), 4.63 (1H, m, H-3β), 3.72 (1H, s, H-7β), 3.68 (2H, ovl, H_2_-23), 1.56 (6H, s, CH_3_-3′ and CH_3_-4’ of clofibric unit), 0.96 (3H, d, *J* = 6.5 Hz, CH_3_-21), 0.91 (6H, ovl, CH_3_-19 and CH_3_-25), 0.67 (3H, s, CH_3_-18). ^13^C NMR (100 MHz, CDCl_3_): δc 173.2, 154.0, 129.3 (2C), 126.8, 120.5 (2C), 79.6, 76.0, 70.6, 60.8, 56.4, 53.4, 50.5, 45.1, 42.8, 41.1, 39.9, 39.5, 38.9, 35.6, 35.0, 33.1, 32.8, 29.6, 28.3, 26.5, 25.5, 25.1, 23.1, 22.1, 20.7, 18.8, 11.7, 11.6. ESI-MS m/z 571.4 [M + H-H_2_O]^+^ and 553.3 [M + H-2H_2_O]^+^.

#### Preparation of compound 5

The product obtained from the reaction of compound **10** (30 mg, 69 mmol) with fenofibric acid was purified by HPLC using a reverse-phase column (Luna C18, 10 μm, 10 mm i.d. x 250 mm) and MeOH/H_2_O 90:10 as eluent (flow rate 3 mL/min) giving compound **5** (8 mg, t_R_ = 21 min).

Compound **5**. Selected ^1^H NMR (400 MHz, CDCl_3_): δ_H_ 7.72 (4H, ovl, H-3″, H-5″, H-2‴ and H-6‴ of fenofibric unit), 7.45 (2H, d, *J* = 8.5 Hz, H-3‴ and H-5‴ of fenofibric unit), 6.88 (2H, d, *J* = 8.8 Hz, H-2″ and H-6” of fenofibric unit), 4.64 (1H, m, H-3β), 3.67 (3H, ovl, H-7β and H_2_-23), 1.67 (3H, s, CH_3_-3′ of fenofibric unit), 1.66 (3H, s, CH_3_-4’ of fenofibric unit), 0.95 (3H, d, *J* = 6.5 Hz, CH_3_-21), 0.90 (6H, ovl, CH_3_-19 and CH_3_-25), 0.67 (3H, s, CH_3_-18). ^13^C NMR (100 MHz, CDCl_3_): δ_C_ 194.5, 173.0, 160.0, 138.3, 136.5, 131.9 (2C), 131.3 (2C), 130.1, 128.4 (2C), 117.3 (2C), 79.6, 76.4, 70.5, 60.9, 56.3, 50.5, 45.2, 42.7, 41.1, 40.0, 39.6, 38.9, 35.6, 35.0, 33.1, 32.9, 29.2, 28.4, 26.4, 25.6, 25.5, 23.7, 23.0, 22.2, 20.7, 18.8, 11.7 (2C). ESI-MS m/z 675.4 [M + H-H_2_O]^+^ and 657.4 [M + H-2H_2_O]^+^.

#### Preparation of compound 8

The product obtained from the reaction of compound **10** (30 mg, 69 mmol) with gemfibrozil was purified by HPLC using a reverse-phase column (Luna C18, 10 μm, 10 mm i.d. x 250 mm) and MeOH/H_2_O 95:5 as eluent (flow rate 3 mL/min) giving compound **8** (5 mg, t_R_ = 24 min).

Compound **8**. Selected ^1^H NMR (400 MHz, CDCl_3_): δ_H_ 7.01 (1H, d, *J* = 7.4 Hz, H-3″ of gemfibrozil unit), 6.66 (1H, d, *J* = 7.4 Hz, H-4″ of gemfibrozil unit), 6.61 (1H, s, H-6″ of gemfibrozil unit), 4.54 (1H, m, H-3β), 3.92 (2H, t, *J* = 5.5 Hz, H-5′ of gemfibrozil unit), 3.68 (3H, ovl, H-7β and H_2_-23), 2.31 (3H, s, CH_3_-8″ of gemfibrozil unit), 2.18 (3H, s, CH_3_-7” of gemfibrozil unit), 1.20 (6H, s, CH_3_-6′ and CH_3_-7’ of gemfibrozil unit), 0.97 (3H, d, *J* = 6.5 Hz, CH_3_-21), 0.92 (6H, ovl, CH_3_-19 and CH_3_-25), 0.68 (3H, s, CH_3_-18). ^13^C NMR (100 MHz, CDCl_3_): δ_C_ 177.4, 157.0, 136.4, 130.2, 123.5, 120.7, 112.0, 74.5, 70.8, 68.1, 60.8, 56.4, 50.5, 45.1, 42.8, 42.0, 41.2, 40.0, 39.5, 39.0, 37.1, 35.6, 35.1, 33.3, 32.9, 29.5, 28.4, 26.6, 25.3, 25.2, 25.0, 23.7, 23.1, 22.2, 21.4, 20.8, 18.8, 15.8, 11.8, 11.7. ESI-MS m/z 607.5 [M + H-H_2_O]^+^ and 589.5 [M + H-2H_2_O]^+^.

### Biological activity assays

#### AlphaScreen on FXR and PPARα of BAR502 hybrids

Compounds’ ability to mediate cofactor recruitment was evaluated by employing AlphaScreen GST Detection Kit (PerkinElmer). Particularly, FXR agonism was evaluated employing 10 nM FXR-LBD GST-fused (Thermo Scientific) and 30 nM of biotinylated SRC1 peptide (CPSSHSSLTERHKILHRLLQEGSPS) in the presence of 20 μg/mL donor and acceptor beads and in buffer containing 50 mM Tris-HCl (pH7.4), 20 mM KCl, 1 mM DTT, and 0.1% BSA. For PPARα assay, a buffer containing 50 mM MOPS, 50 mM NaF, 0.05 mM CHAPS, and 0.05% BSA (pH = 7.4) was used, and 20 nM PPARα LBD GST fused (Thermo Scientific) and 60 nM of biotinylated SRC1 peptide were employed. Both acceptor and donor beads were added to reach 20 μg/mL final concentration. Incubations were performed in a final volume of 25 μL employing 384 wells Optiplate and Alpha signal was measured with Envision 2,105 (Perkin Elmer) multimode plate reader. For efficacy, compounds were tested at 10 μM for FXR and 50 μM for PPARα.

#### Transactivation assay

HepG2 and HEK-293T cells were cultured at 37 °C in minimal essential medium (MEM) and Dulbecco’s Modified Eagle Medium (DMEM) respectively, supplemented with 10% fetal bovine serum, 1% L-glutamine, 100 U/mL penicillin, and 100 μg/mL streptomycin. To investigate FXR-mediated transactivation, the HepG2 cells were transfected with 200 ng of the reporter vector p (hsp27)TK-Luc containing the FXR response element IR1 cloned from the promoter of heat shock protein 27 (hsp27), plus 100 ng pSG5-FXR, 100 ng pSG5-RXR, and 100 ng pGL4.70 Renilla. To evaluate GPBAR1 mediated transactivation, HEK-293T cells were transfected with 200 ng of human pGL4.29 (Promega), a reporter vector containing a cAMP response element (CRE) that drives the transcription of the luciferase reporter gene luc2P, with 100 ng of pCMVSPORT6-human GPBAR1, and with 100 ng of pGL4.70. At 24 h post-transfection, cells were stimulated 18 h with 10 μM TLCA (taurolithocholic acid a GPBAR1 agonist) or CDCA (FXR agonist) and compounds. To calculate the EC_50_ on FXR and GPBAR1, dose-response curves were performed in HEK-293T cells transfected as described above and then treated with increasing concentrations of compounds (from 0.1 to 50 µM). After treatments, cells were lysed in 100 μL of lysis buffer (25 mM Tris-phosphate, pH 7.8; 2 mM DTT; 10% glycerol; 1% Triton X-100), and 10 μL of cellular lysate was assayed for luciferase activity using the Firfly &Renilla Luciferase assay kit (Biotium). Luminescence was measured using Glomax 20/20 luminometer (Promega). Luciferase activities were assayed and normalized with Renilla activities.

#### Cell culture

U937 cell line were cultured in RPMI 1640 supplemented with 10% FBS, 1% glutamine, and 1% penicillin/streptomycin and were regularly passaged to maintain exponential growth. After 24 h of starvation cells were classically activated with LPS (100 ng/mL; Sigma-Aldrich, St. Louis, MO), and exposed or not to **BAR502**, clofibrate and compound **1** at the concentration of 5 and 10 μM for 18 h. At the end of the experiment the cells were recovered.

#### 3T3-L1 cell differentiation

The 3T3-L1 cells were cultured in Dulbecco’s modified Eagle’s medium (DMEM; Euroclone), supplemented with 10% fetal bovine serum, 1% l-glutamine, 100 U/mL penicillin, and 100 μg/mL streptomycin, at 37 °C. Preadipocytes of 3T3-L1 cells were plated at 1.7 × 10^6^ cells in T75 flasks, and grown to confluence. Two days after reaching confluence (day 0), the cells were stimulated with differentiation medium (DIM), either alone (DMEM, 10% FBS, 500 μM 3-isobutyl-1-methylxanthine, 1 μM dexamethasone, 1 mg/mL insulin), or in combination with **BAR502** and clofibrate at the concentration of 5 µM or with compound **1** (10 µM). On day 3, the DIM was replaced by insulin medium (DMEM, 10% fetal bovine serum, 1 mg/mL insulin) alone or with the compounds. On day 7 cells were recovered.

#### RNA isolation and qRT-PCR

Total mRNA extraction from U937 and 3T3.L1 was performed using Tri-Reagent (Zymo Research) and Direct-zol™ RNA MiniPrep w/Zymo-Spin™ IIC Columns (Zymo Research, Irvine, CA). After purification from genomic DNA using DNase I (Thermo Fisher Scientific, Waltham, MA), 1 μg of RNA from each sample was reverse transcribed using Kit FastGene Scriptase Basic (Nippon Genetics, Mariaweilerstraße, Düren, Germaniain) in 20-μL of reaction volume; 50 ng of cDNA was amplified in a 20-μL solution containing 200 nM each primer and 10 μL of PowerUp™ SYBR™ Green Master Mix (Thermo Fisher Scientific, Waltham, MA). All reactions were performed in triplicate using the following thermal cycling conditions: 3 min at 95°C, followed by 40 cycles of 95°C for 15 s, 56°C for 20 s, and 72°C for 30 s, using a QuantStudio 3 system (Applied Biosystems, Foster City, CA). The relative mRNA expression was calculated accordingly to the ΔCt method. Primers were designed using the software PRIMER3 (http://frodo.wi.mit.edu/primer3/) using published data obtained from the NCBI database. The primer used were as following (forward and reverse): hGAPDH (for CAG​CCT​CAA​GAT​CAT​CAG​CA; rev GGT​CAT​GAG​TCC​TTC​CAC​GA), hIl-1β (for GTG​GCA​ATG​AGG​ATG​ACT​TG; rev GGA​GAT​TCG​TAG​CTG​GAT​GC), hIL6 (for AGT​GAG​GAA​CAA​GCC​AGA​GC; rev CAG​GGG​TGG​TTA​TTG​CAT​CT) hCXCL2 (for GCA​GGG​AAT​TCA​CCT​CAA​GA; rev GAC​AAG​CTT​TCT​GCC​CAT​TC) hTNFα; (for AGC​CCA​TGT​TGT​AGC​AAA​CC; rev TGA​GGT​ACA​GGC​CCT​CTG​AT), mAdiponectin (for ATG​CAG​GTC​TTC​TTG​GTC​CT; rev GAG​CGA​TAC​ACA​TAA​GCG​GC), mPparɤ (for GCC​AGT​TTC​GAT​CCG​TAG​AA; rev AAT​CCT​TGG​CCC​TCT​GAG​AT), mCebp (for ATT​TCT​ATG​AGA​AAA​GAG​GCG​TAT​GT; rev AAA​TGT​CTT​CAC​TTT​AAT​GCT​CGA​A).

#### Chemical and metabolic profiling of compound 1

Chemical stability in HCl Buffer, PBS buffer and culture medium (EMEM) was evaluated by LC-MS dissolving compound **1** at 10 μM (final concertation) in cited buffers and injecting aliquots removed from incubations at different time points. Microsomal stability was evaluated employing 50 mM potassium phosphate buffer (pH 7.4) containing 0.15 mg of Human liver microsomes (Sigma-Aldrich, St. Louis, MO, United States) 5 mM MgCl_2_, 1 mM NADPH, 5 mM glucose 6-phosphate, 0.4 U·mL^−1^ glucose 6-phosphate dehydrogenase. Compounds were tested at 1 µM final concentration. Aliquots, removed at different time points, were diluted with ice-cold acetonitrile and analyzed by LC-MS.

Cryopreserved HEP10 Pooled Human Hepatocytes were thawed in 37°C water bath for 2 min and then transferred into CHRM^®^ Medium. After centrifugation at room temperature cells were resuspended in pre-warmed Incubation Medium and diluted to correct seeding density (5 × 105 cells/mL). Hepatocytes plated in a 24-well plate were treated with compound **1**, **BAR502** or clofibrate, and aliquots of cell suspension withdrawn at different time points (0, 15′, 30’, 1, 2, 4, 6 and 8 h) were extracted with acetonitrile and analyzed by LC-MS in MRM mode. Testosterone and 6-ECDCA (6a-ethyl-chenodeoxycholic acid) were employed as controls.

#### In cell permeation and metabolism of compound 1

HepG2 cells were treated with compound **1**, **BAR502** or clofibrate. At different time points (0–480 min) culture mediums and cells pellets were collected, extracted by adding ice-cold acetonitrile and analyzed by LC-MSMS.

#### Compound 1 metabolite quantization *in vivo*

25 μL of plasma from mice treated with compound **1** or clofibrate (30 mg/kg) were collected at various time points after administration (0, 1, 3, 6, 8 h), extracted adding 75 μL of acetonitrile, vortexed and centrifuged at 15100 *g* for 10 min. Supernatants were analyzed in MRM mode (Agilent 1,290 Infinity II coupled with Agilent 6470 TQ). Transitions have been optimized for each analyte and the amount of different metabolites was evaluated employing an in matrix calibration curve.

Separation was achieved by means of a linear gradient H_2_O with 0.1% formic acid and acetonitrile with 0.1% formic acid employing a Kinetex C18 (100 × 2.1 2.6um-Phenomenex).

## Results

### Chemistry

#### Synthesis of BAR502-fibrate hybrids

BAR502-fibrate derivatives (**1**–**9**) were obtained through the coupling reaction of the hydroxyl groups of **BAR502** (6α-ethyl-3α,7α-dihydroxy-24-nor-5β-cholan-23-ol) with different fibric acids (fenofibric acid, clofibric acid or gemfibrozil) carried out using EDC HCl/HOBt/DIPEA in DMF.

**SCHEME 1:**
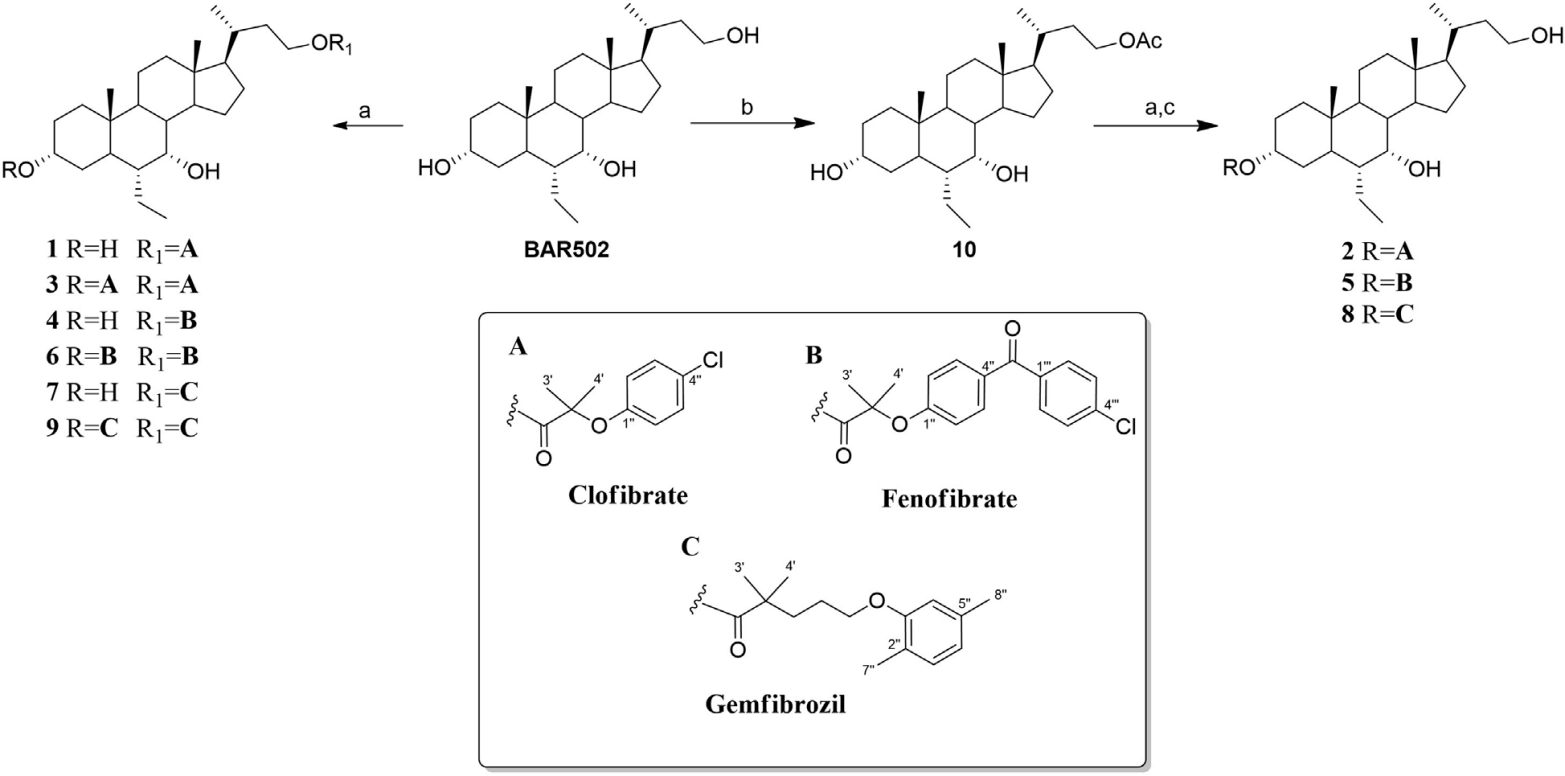
Reagents and conditions: **(A)** Fibric acids **(A–C)**, EDC HCl (2 eq), HOBt (2 eq), DIPEA (4 eq) in dry DMF; **(B)** Ac_2_O in dry pyridine a 0°C; **(C)** *p*-TsOH in CHCl_3_: MeOH.

The hydroxyl groups at positions C-3 and C-23 of **BAR502** can be easily conjugated, whereas the hydroxyl group at C-7 is not accessible due to steric hindrance. The chromatographic purification afforded BAR502-fibrate derivatives (**1**, **3**, **4**, **6**, **7** and **9**) characterized by one or two fibric units at C-23 and at C-3/C-23 positions, respectively (Scheme 1).

Regioselective conjugation at C-3 position was then performed preceding the coupling reaction with acetylation at C-23 hydroxyl group of **BAR502** (compound **10**) followed by deacetylation to obtain compounds **2**, **5** and **8** (Scheme 1).

### Biological activity

#### Biological evaluation of BAR502-fibrate hybrids (**1–9**)

As previously reported, **BAR502** is equally potent toward both the FXR and GPBAR1 receptors with EC_50_ for FXR ≈2 μM and EC_50_ for GPBAR1 ≈ 400 nM (Festa et al., 2014; Cipriani et al., 2015). Therefore, derivatives **1-9** were tested towards GPBAR1, FXR and PPARα (Table 1), the pharmacological targets of original molecules, BAR502 and fibrates. In detail, the activity towards GPBAR1 was evaluated in the luciferase reporter assays on HEK-293T cells transiently transfected with the membrane bile acid receptor GPBAR1, and the recruitment of the coactivator SRC-1 through Alpha screen technology (Mukherjee et al., 2002) was employed to assess their activity towards FXR and PPARα. CDCA, TLCA and fenofibrate were used as positive control at 10 µM and their effects were defined as 100%. Of interest, the results shown in Table 1 demonstrate that compound 1 is a dual FXR/GPBAR1 agonist with FXR efficacy of 79% and GPBAR1 efficacy of 145% when compared to CDCA and TLCA, the most potent endogenous FXR and GPBAR1 agonists, respectively. Compound **3**, conjugated with clofibrate units at both C-3 and C-23, is a preferential GPBAR1 agonist (efficacy of 101% vs. TLCA) with a low activity toward FXR (efficacy of 48%). All compounds are inactive towards PPARα, except **BAR502** that showed a slightly activity (efficacy of 36%).

**TABLE 1:** Efficacy towards FXR and GPBAR1 of compounds **1**-**9**

Compound	FXR efficacy^a^ (% vs*.* CDCA)	GPBAR1 efficacy^b^ (% vs*.* TLCA)	PPARα efficacy^a^ (% vs. fenofibrate)
**BAR502**	153.0 ± 3.1	79.5 ± 11.8	36 ± 12
**1**	79.0 ± 3.8	145 ± 10	n.a
**2**	3.9 ± 0.1	46 ± 16	n.a
**3**	48.0 ± 5.5	101.0 ± 0.4	27 ± 10
**4**	14.0 ± 1.8	41.0 ± 3.0	13 ± 3
**5**	n.a	40.0 ± 0.7	6.6 ± 1.9
**6**	n.a	30 ± 17	1.7 ± 0.5
**7**	4.1 ± 0.6	14.0 ± 1.3	22 ± 18
**8**	n.a	29.4 ± 5.1	5.3 ± 0.8
**9**	5.1 ± 1.9	28.5 ± 0.4	n.a

^a^ AlphaScreen coactivator recruitment assay measuring direct interaction between FXR, or PPARα, and SRC-1; compounds were tested at 10 μM for FXR, and 50 mM for PPARa.

^b^ Transactivation assays on HEK-293T, cells. Results are expressed as mean ± SD. n.a. = not active.

The above data agree with previous reports bringing to our attention the pharmacophoric rule of the 3α-hydroxyl group in FXR binding and affirming the side chain of cholane scaffolds as more amenable to modification without affecting FXR activity. This trend was also confirmed by the results on compound **2**, the mono-substituted derivative at C-3, with a complete loss in FXR activity. The mono-substitution at C-3 was also detrimental to the activity on GPBAR1. Functionalization with larger units such as gemfibrozil and fenofibrate leads to a considerable loss of activity towards GPBAR1 (efficacies less than 41%) and a complete inactivity toward FXR (Table 1).

The concentration-response curve on dual FXR and GPBAR1 obtained by a transactivation assay (Table 2) confirmed compound **1** as a potent dual FXR/GPBAR1 agonist (EC_50_ 2.8 and 0.37 μM, respectively) and compound **3** as a preferential GPBAR1 agonist (EC_50_ 25 μM on FXR vs*.* 1.8 μM on GPBAR1).

**TABLE 2:** EC_50_ values for compounds **1** and **3**

Compound	FXR EC_50_ ^a^	FXR EC_50_ ^b^	GPBAR1 EC_50_ ^b^
**BAR502**	0.69 ± 0.15	2.0 ± 0.2	0.4 ± 0.1
**1**	3.4 ± 1.2	2.80 ± 0.01	0.37 ± 0.02
**3**	17.9 ± 2.6	25.0 ± 0.01	1.8 ± 0.35

^a^ EC_50_ measured by AlphaScreen coactivator recruitment assay measuring a direct interaction of FXR, with SRC-1; compounds were tested at 10 μM for FXR.

^b^ Transactivation assays on HepG2 cells or HEK-293T, cells. EC_50_ values (μM) were calculated from at least three experiments. Results are expressed as mean ± SD.

Collectively, conjugation of **BAR502** with clofibric acid provided the hybrid compound **1**, which retains dual activity towards FXR/GPBAR1 and can generate, as a result of hydrolysis in a biological environment, **BAR502**, with FXR/GPBAR1 dual activity, and clofibric acid with agonistic activity on PPARα, allowing simultaneous effect on three receptors (FXR/GPBAR1/PPARα). In addition, the shift of activity of compound **3**, which maintains a good potency toward GPBAR1 with respect to **BAR502**, yielded a selective GPBAR1 agonist.

**FIGURE 2:**
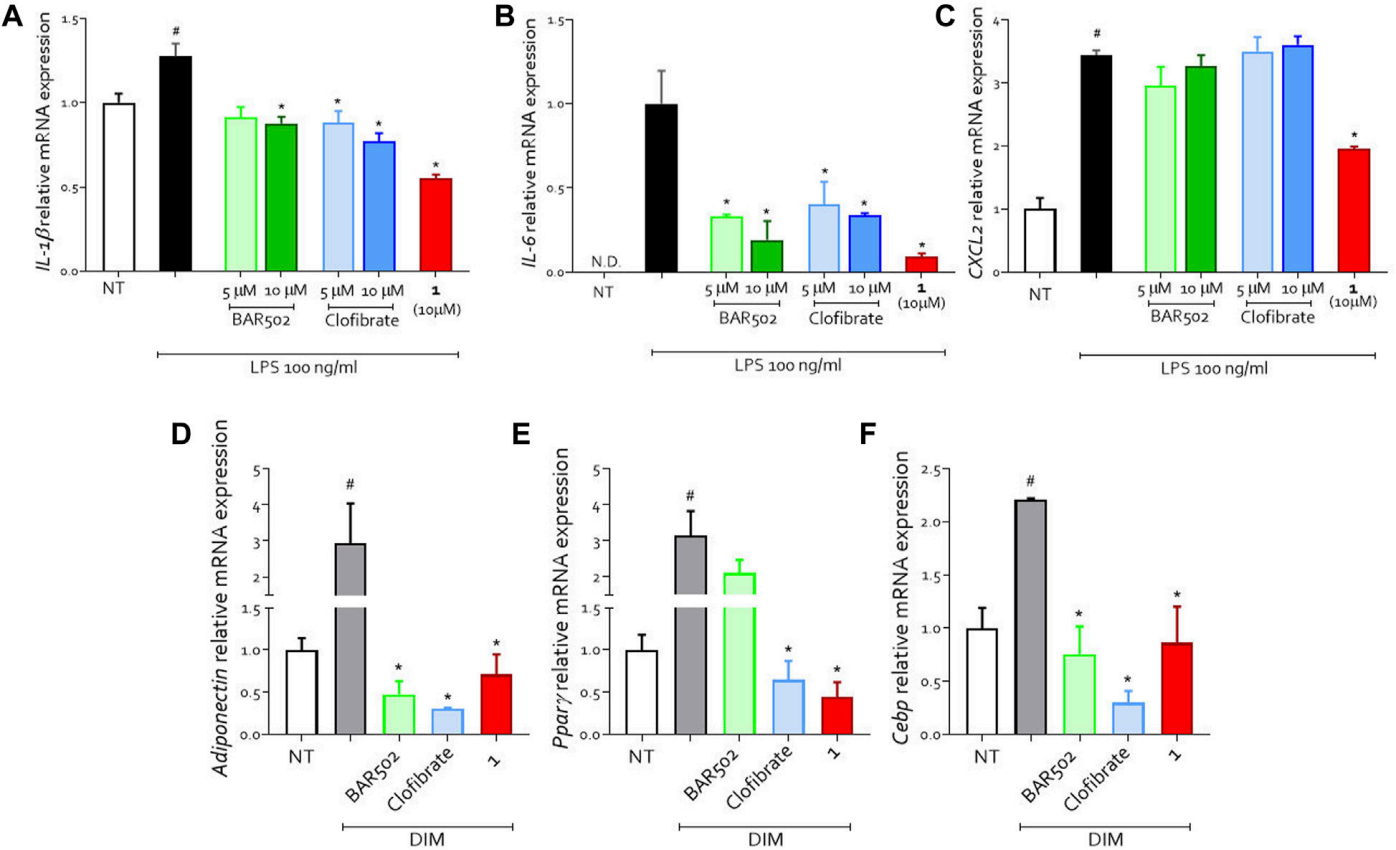
U937 cells were classically activated with LPS (100 ng/mL) and exposed or not to **BAR502**, clofibrate (5 and 10 μM) and compound **1** (10 μM) for 18 h. Quantitative real-time PCR analysis of expression of pro-inflammatory genes **(A)** IL-1β; **(B)** IL-6; **(C)** CXCL2; ND: not detectable. Data are normalized to GAPDH. Results represent mean ± SEM, #*p* < 0.05 vs. NT, *p < 0.05 vs. LPS group. **(D–F)** Effects on differentiation-related gene expression. Preadipocyte 3T3L1 cells were differentiated for 7 days in the presence of differentiation medium (DIM) alone or in combination with **BAR502** and clofibrate at the concentration of 5 µM or with compound **1** (10 µM). Quantification of relative mRNA expression of adipogenic marker genes: **(D)** Adiponectin, **(E)** Pparγ and **(F)** Cebp. Data are means ± SEM, #*p* < 0.05 versus NT; *p < 0.05 versus differentiated cells (DIM). Data are normalized to Gapdh mRNA expression.

We carried out an *in vitro* study on U937 cell line (a human monocyte/macrophage cell line) and 3T3.L1 cell line (a murine pre-adipocyte cell line) with compound **1** to investigate the anti-inflammatory and metabolic properties of the new compound (Figure 2). We first stimulated U937 cells with LPS (100 ng/mL) which induces a bias towards a pro-inflammatory phenotype as shown by the upregulation of IL-1β, IL-6 and CXCL2 (Figures 2A–C). The cells were treated with **BAR502**, clofibrate and compound **1**. All molecules showed anti-inflammatory activity by reducing the expression of pro-inflammatory cytokines (Figures 2A, B). However, compound 1 showed the greatest efficacy in reducing the expression of both IL-1β and IL-6 (Figures 2A, B) and was the only compound also reducing the expression of the chemokine CXCL2 (Figure 2C). Collectively these data underlined the potent anti-inflammatory effect of compound **1**.

To analyse the metabolic effects, we treated a pre-adipocyte cell line (3T3.L1) with a differentiation medium (DIM) alone or in combination with **BAR502**, clofibrate or compound **1** (Figures 2D–F). DIM-induced adipocyte differentiation as shown by upregulation of Adiponectin, Pparγ and Cebp expression (Figures 2D–F).

Compound **1** counteracted the effect of DIM as indicated by the reduction in the expression of all markers of adipocyte differentiation (Figures 2D–F).

#### Metabolic profiling of compound **1**

In order to decipher the pharmacological behavior of compound **1**, the stability of **1** was evaluated in several chemical environments before proceeding in more complex biological systems. Chemical stability was assessed by LC-MS and compound **1** resulted stable in HCl buffer for 2h, in PBS buffer at pH 7.4 and in culture medium for 8 h.

For the analysis of *in vivo* biotransformation, plasma from mice treated with compound **1** was analyzed by LC-MSMS. In negative ion mode, we detected clofibric acid as expected, and a species with *m/z* 405.3 [M-H]^-^, 14 mass units higher than that of **BAR502**.

The ionization ability in negative ion mode suggested the carboxylic acid nature of the metabolite, deriving probably from an oxidative process of the primary hydroxyl group at C-23 of **BAR502**. In addition, in the positive ion mode, this species showed two main ions with *m/z* 389.2 [M-H_2_O]^+^ and 371.3 [M-2H_2_O]^+^ amenable to the ion-source loss of water molecules, typical ionization pattern of the parent **BAR502**. The MS^3^ spectrum showed a pattern of fragmentation similar to **BAR502,** compatible with the supposed metabolite (Supplementary Figure S1). The carboxylic acid derivative of **BAR502**, named **BAR505** (Festa et al., 2014), available in our laboratory, was used as analytical standard, allowing us to confirm the identity of putative metabolite and to perform further analysis.

**FIGURE 3:**
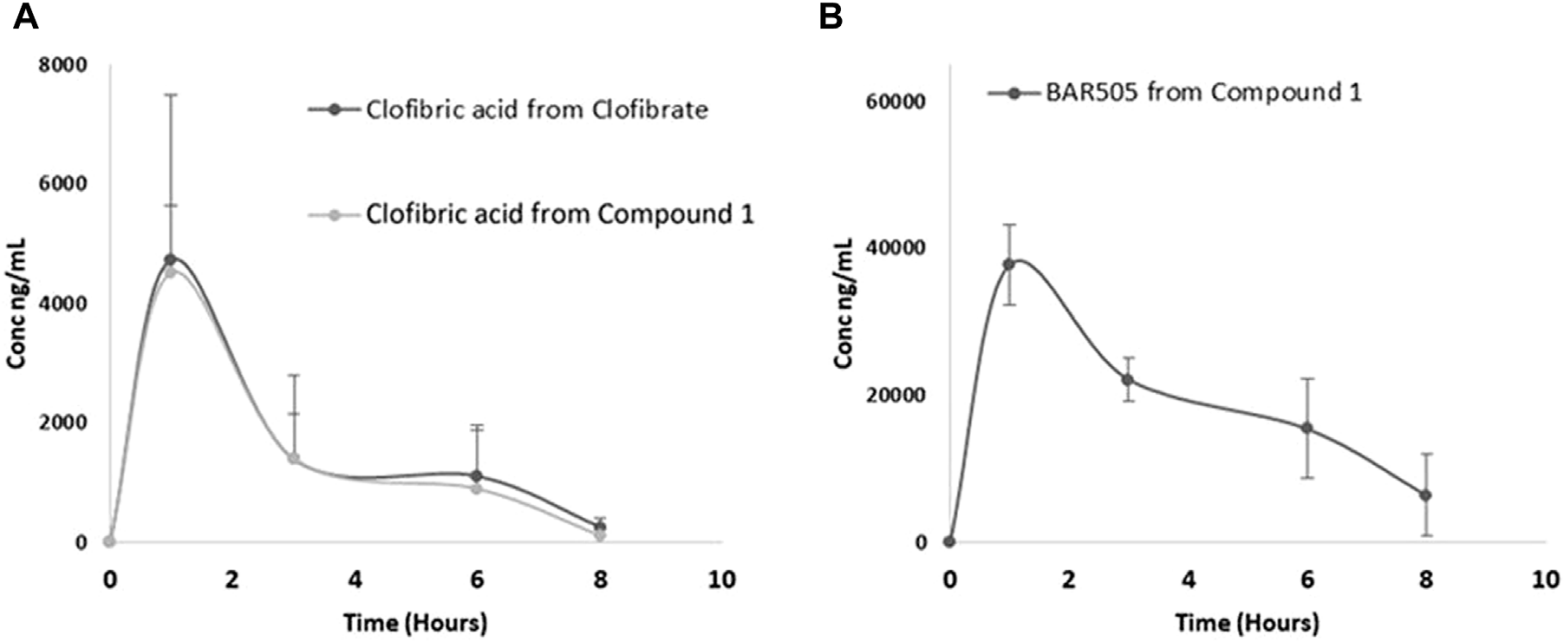
Plasma profile in mice treated with compound **1** or clofibrate: **(A)** clofibric acid detected from clofibrate and compound **1**; **(B)** **BAR505** detected from compound **1**.

Subsequently, we quantitatively evaluated the plasma profile of compound **1** in mice treated with either compound **1** or clofibrate. Clofibric acid, **BAR502**, **BAR505** and compound **1** were quantified using an MRM MS method (Figure 3). Compound **1** was not found in plasma samples; its hydrolysis product, **BAR502**, was exclusively detected at T = 1 h (68.8 ± 24.3 nM), whereas the highly abundant species was the oxidized metabolite **BAR505**. Concerning clofibric acid release, the quantity generated from the hydrolysis of **1** compared with the one obtained from clofibrate was superimposable, although the lower molecular weight of the latter, suggesting that compound **1** is likely a good pro-drug.

Since **BAR505** represents the main circulating metabolite of **BAR502**, we performed transactivation assays demonstrating that **BAR505** preserved agonistic activity on FXR (efficacy vs*.* CDCA 457%) and GPBAR1 (efficacy vs*.* TLCA of 103.6%) in dose-dependent manner, with EC_50_ of 1.9 ± 0.14 and 3.2 ± 1.1 μM, respectively, comparable to the activity of the precursor compound **BAR502** (Supplementary Figure S2). Moreover, **BAR505** activity on PPARα was assayed through Alfa screen and resulted slightly active (efficacy vs*.* fenofibrate of 40%), similarly to **BAR502**.

**FIGURE 4:**
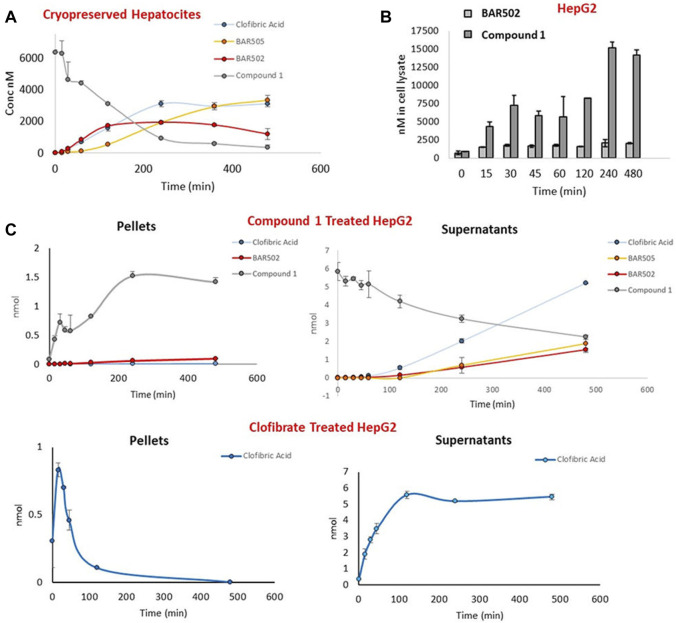
**(A)** Time course concentration of compound **1**, clofibric acid and **BAR502** as hydrolysis products, and **BAR505** in cryopreserved hepatocytes; **(B)** Concentration of **BAR502** and compound **1** in pellets of HepG2 cells treated with **BAR502** and compound 1 respectively; **(C)** Comparison of compound **1** and clofibrate chemical behavior in cell pellets and supernatants of HepG2 cells measuring the concentration of compound **1**, clofibric acid and **BAR502** as hydrolysis products, and **BAR505**.

The next step was the study of the behavior of the hybrid compound **1** in human cellular and enzymatic systems such as microsome, cryopreserved hepatocyte and HepG2 cells, in order to evaluate respectively compound **1** stability towards metabolization, and its ability to penetrate the cell membrane (Figure 4).

Compound **1** showed a t_1/2_ value of 27 min (Cl_int_ = 87) in human microsomes, notably higher than clofibrate that is readily converted in clofibric acid within 30 min (Supplementary Figure S3).

The enzymatic stability of compound **1** was further demonstrated in cryopreserved hepatocytes, measuring, by LC-MSMS, the amounts of compound **1**, clofibric acid, **BAR502** and **BAR505** (Figure 4A). The results revealed that compound **1** was stable also in this model with a t_1/2_ of 121 min.

Finally, the ability to penetrate cell membrane was investigated in HepG2 cells treated with compound **1**, or **BAR502** or clofibrate. At several time points (0–480 min), culture mediums and cells pellets were collected and analyzed by LC-MSMS (Figures 4B, C). As shown in Figure 4C, compound **1** reached considerable amount in cell pellets, showing a higher cell permeation compared to **BAR502**.

Moreover, the comparison of the amount of clofibric acid, generated by compound **1** with that generated from clofibrate, suggested that the hybrid molecule was hydrolyzed more slowly, probably due to its higher steric hindrance (Figure 4C). On the contrary, in clofibrate treated cells, clofibric acid quickly reaches a peak of concentration in cell pellets and successively accumulates in supernatants.

## Discussion

NAFLD is a condition characterized by the accumulation of fat in the liver and it is considered the hepatic manifestation of the metabolic syndrome (Pawlak et al., 2015). Its pathogenesis has not been fully elucidated, but, within the involvement of different organs and systems, crucial role is played by inflammatory mediators, especially those deriving from the adipose tissue and the liver, which are involved in the development of necroinflammation and fibrosis.

Fibrates, a class of hypolipidemic drugs able to activate peroxisome proliferator-activated receptor α (PPARα), were proven to be an effective treatment for patients with obesity, hyperlipidemia, and NAFLD. These drugs featured liver beneficial effects including improvement of fibrosis, inflammation, and hepatic lipid homeostasis by activating PPARα (Romero et al., 2020).

On the other hand, we have recently identified **BAR502**, a semisynthetic bile acid able to act as a dual FXR and GPBAR1 ligand, as a valuable candidate in the treatment of NASH. This compound exhibited protective effects in a mouse model of NASH induced by a high-fat diet (HFD), such as improving adipose function with browning of white adipose tissue and reducing liver fibrosis and steatosis (Carino et al., 2017).

In this paper, we propose the development of hybrid compounds potentially useful for the treatment of metabolic diseases, obtained by conjugation of **BAR502** with three different fibrates via ester bond, with the aim to evaluate the ability to be hydrolyzed in physiologic fluids releasing two active chemical entities, with synergistic action in the treatment of NAFLD. Within the library of hybrids, compound **1** showed an interesting activity, maintaining a similar **BAR502** dual behavior towards FXR and GPBAR1 receptors. Furthermore, the new hybrid compound showed anti-inflammatory activity, reducing the expression of IL-1β, IL-6 and CXCL2, and was able to reduce the expression of all markers of adipocyte differentiation in 3T3.L1 cells.

The conjugation of **BAR502** with clofibric acid revealed high stability in several environments. The potential hydrolysis pattern of compound **1** was evaluated in more complex systems, such as plasma and liver cells, evidencing the hydrolysis and metabolization in mice to clofibric acid and to the oxidized product **BAR505**. Importantly, **BAR505** maintains a full dual agonistic activity toward FXR and GPBAR1, comparable to the parent compound **BAR502**.

We have considered the behavior of hybrid compound **1** in human cryopreserved hepatocytes, a fully competent metabolizing system. The hybrid compound was found to be stable in this model, with a t_1/2_ of 121 min, and its hydrolysis and oxidation of **BAR502** to carboxylic acid were further confirmed and quantified in this system. The employment of HepG2 cells allowed us to appreciate a higher membrane permeability of the hybrid compound compared to **BAR502**.

These data showed that compound **1** reached considerable amount in cells with a higher cell permeation score compared to **BAR502**. In addition, we proved that compound **1** releases quantity of clofibric acid similarly to clofibrate, despite the higher molecular weight of compound **1**, leading additionally the release of the active **BAR505**.

We evaluated the oral bioavailability of compound **1** and clofibrate in mice, quantifying clofibric acid, **BAR502**, **BAR505** and the unmodified compound **1** in plasma. While the hybrid compound was not detected in plasma samples, **BAR505** represents the main circulating form of **BAR502**. These data, together with the high cell permeability shown by compound **1** in HepG2 cells, suggested that compound **1** could be considered a promising prodrug, characterized by an increased uptake and with consequent higher plasmatic concentrations of products of hydrolysis (clofibric acid) and oxidation (**BAR505**).

## Data Availability

The original contributions presented in the study are included in the article/Supplementary Material, and further inquiries can be directed to the corresponding author without undue reservation.
