# Supplementary material for: BAR502/fibrate conjugates: synthesis, biological evaluation and metabolic profile
Source: Front Chem. 2024 Jul 17;12:1425867. doi: 10.3389/fchem.2024.1425867 (PMC11289669; doi:10.3389/fchem.2024.1425867)

Supplementary Material

BAR502/fibrate conjugates: synthesis, biological evaluation and metabolic profile

Claudia Finamore[1], Simona De Marino[1], Chiara Cassiano[1], Giuliano Napolitano[1], Pasquale Rapacciuolo[1], Silvia Marchianò[2], Michele Biagioli[2], Rosalinda Roselli[2], Cristina Di Giorgio[2], Carmen Festa[1*], Stefano Fiorucci[2], and Angela Zampella[1]

Figure S1. MS^3 spectra of **BAR505** (metabolite and standard) and **BAR502** S2
Figure S2. Dose/response curves of **BAR505** on FXR and GPBAR1 S2
Figure S3. Comparison of compound **1** and clofibrate stability in microsome S3
Figures S4-5. ^1H and ^{13}C NMR spectra of compound **1** S4
Figures S6-7. ^1H and ^{13}C NMR spectra of compound **2** S5
Figures S8-9. ^1H and ^{13}C NMR spectra of compound **3** S6
Figures S10-11. ^1H and ^{13}C NMR spectra of compound **4** S7
Figures S12-13. ^1H and ^{13}C NMR spectra of compound **5** S8
Figures S14-15. ^1H and ^{13}C NMR spectra of compound **6** S9
Figures S16-17. ^1H and ^{13}C NMR spectra of compound **7** S10
Figures S18-19. ^1H and ^{13}C NMR spectra of compound **8** S11
Figures S20-21. ^1H and ^{13}C NMR spectra of compound **9** S12

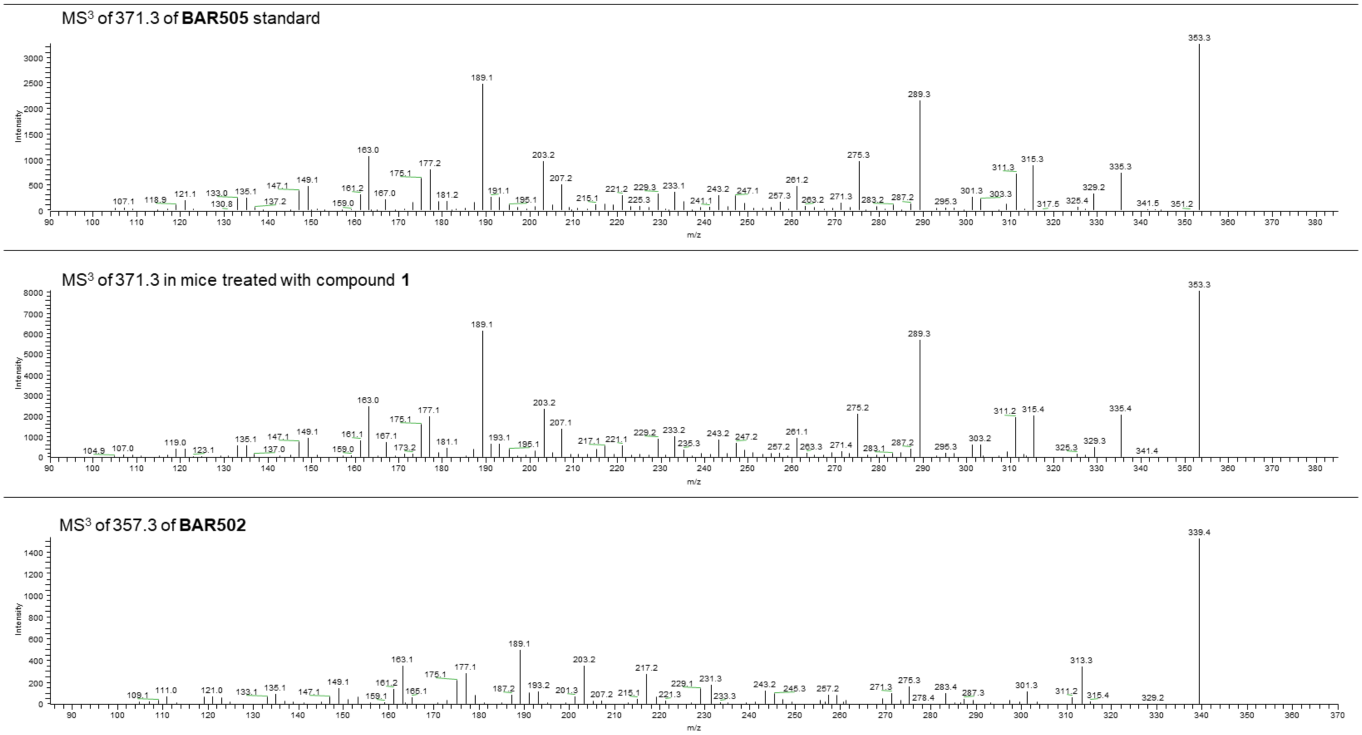

Figure S1. MS^3 spectra of **BAR505** standard, **BAR505** from plasma of mice treated with compound **1** and **BAR502**.

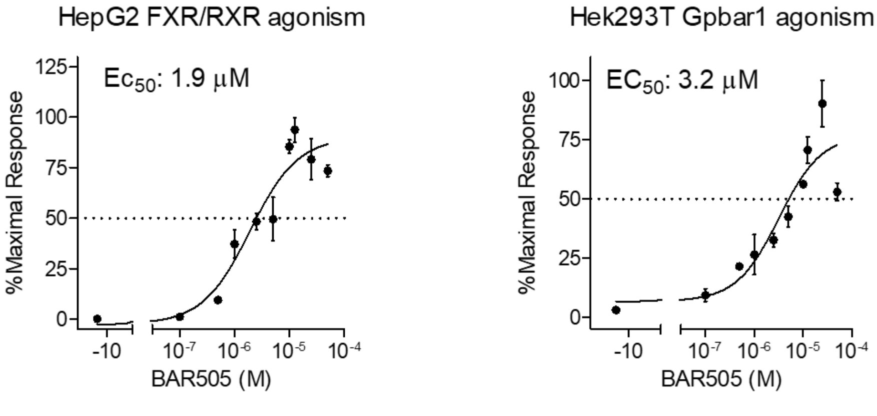

Figure S2. *In vitro* evaluation of **BAR505** on FXR (left) and on GPBAR1 (right) in transactivation assays.

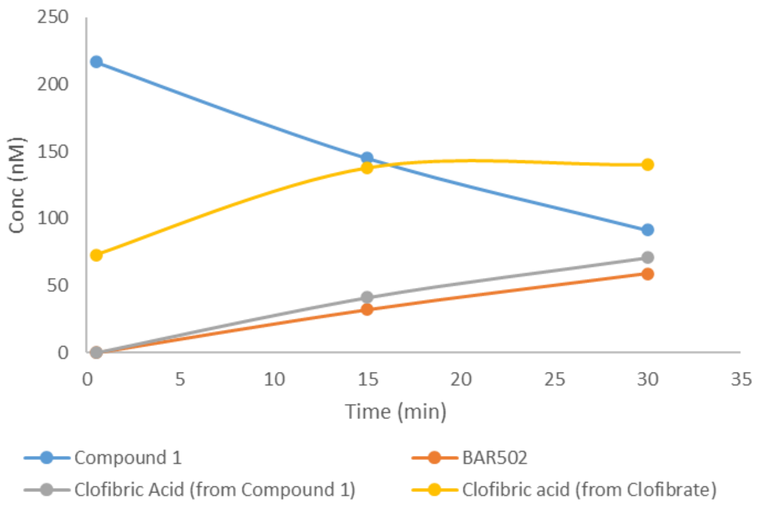

Figure S3. Stability evaluation in microsome of compound **1** and clofibrate and comparison of clofibric acid release.

Figure S4. ^{1}H NMR of compound **1** (700 MHz, $CDCl_3$)

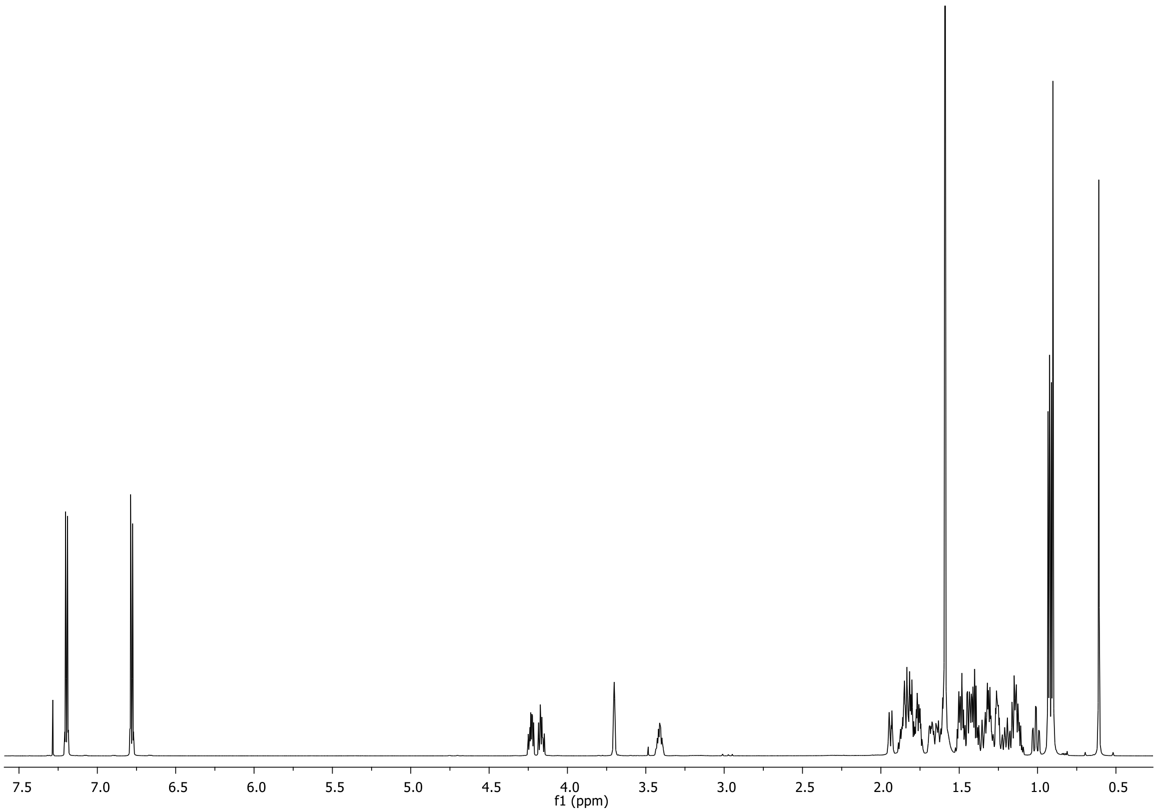

Figure S5. ^{13}C NMR of compound **1** (100 MHz, $CDCl_3$)

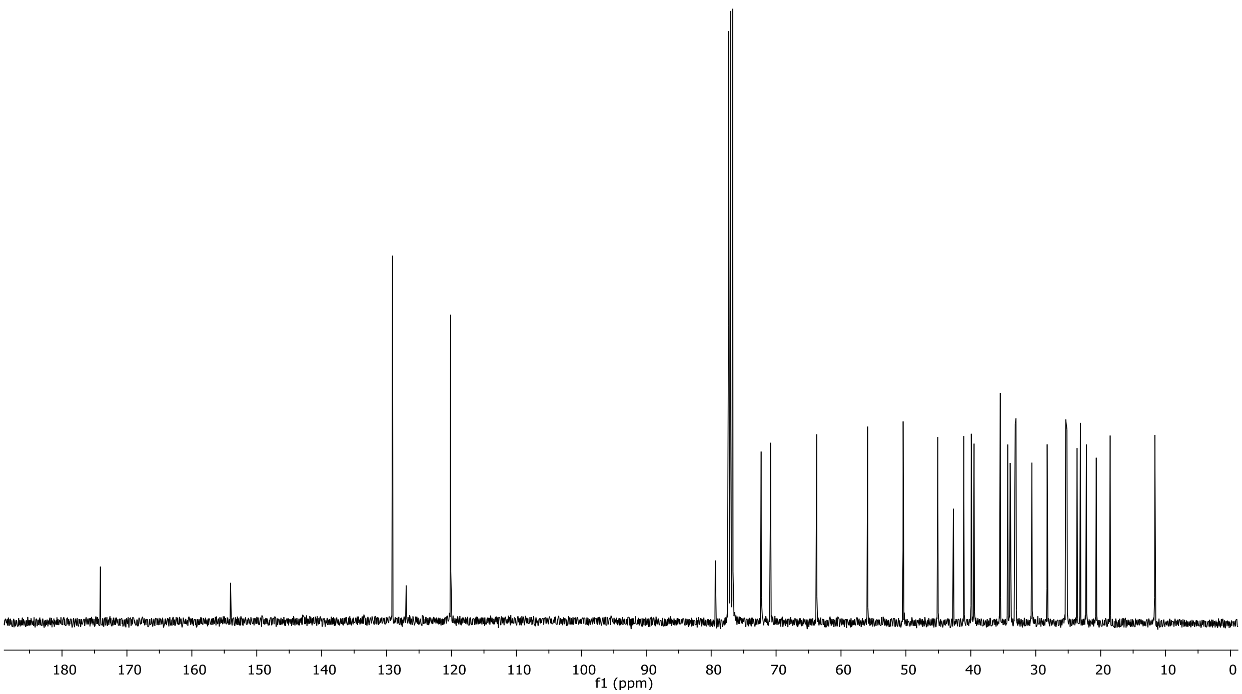

Figure S6. ^{1}H NMR of compound **2** (400 MHz, $CDCl_3$)

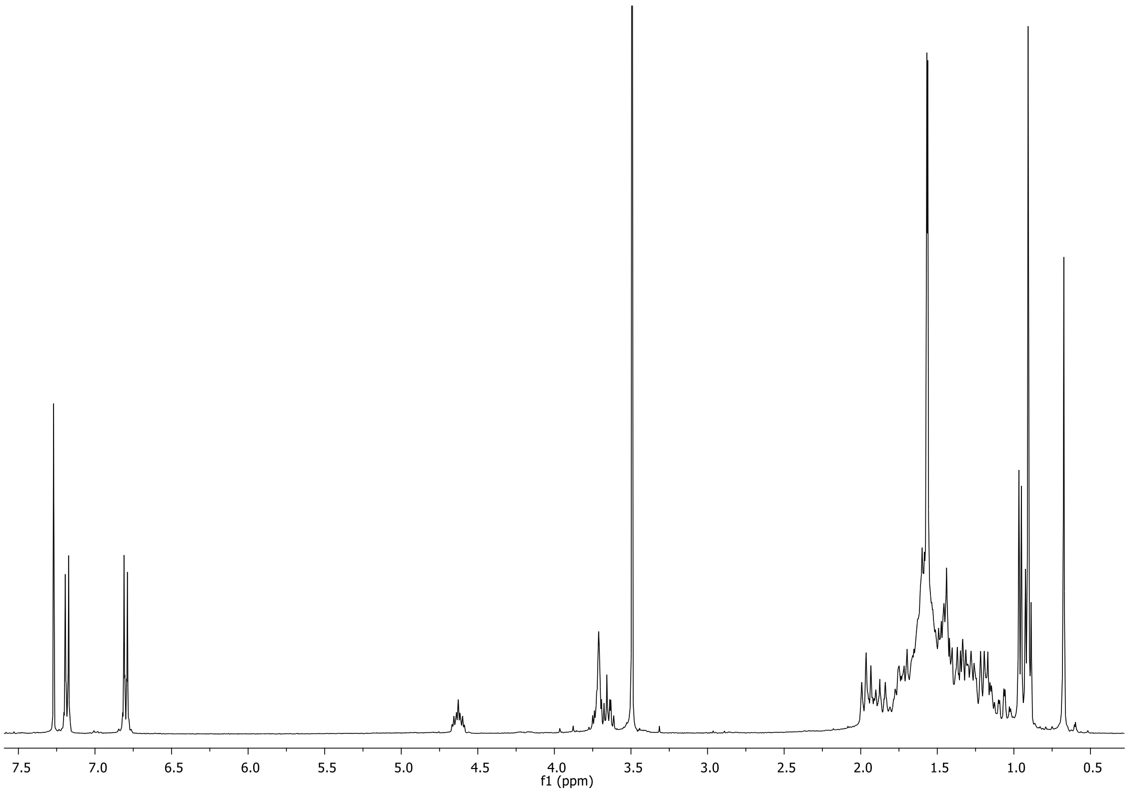

Figure S7. ^{13}C NMR of compound **2** (100 MHz, $CDCl_3$)

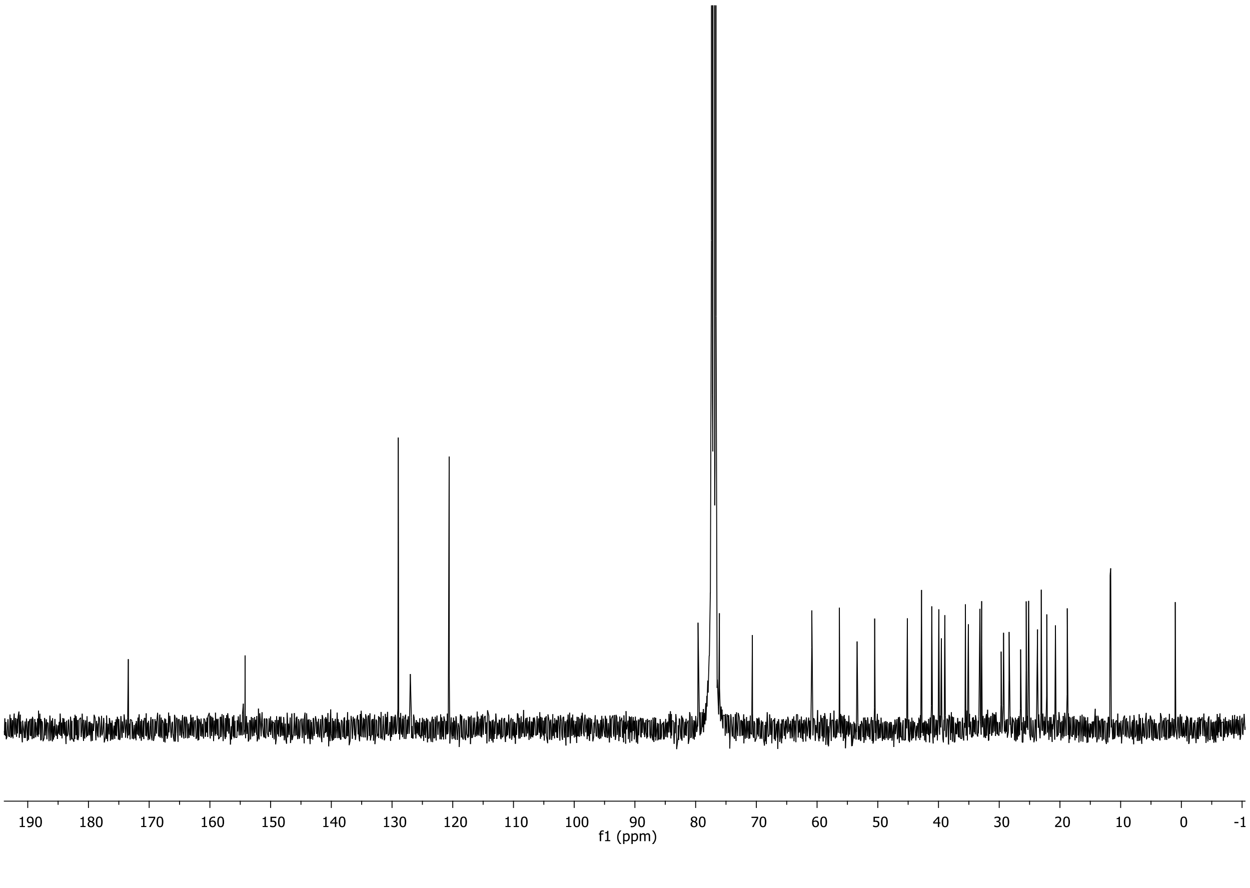

Figure S8. ^{1}H NMR of compound **3** (400 MHz, $CDCl_3$)

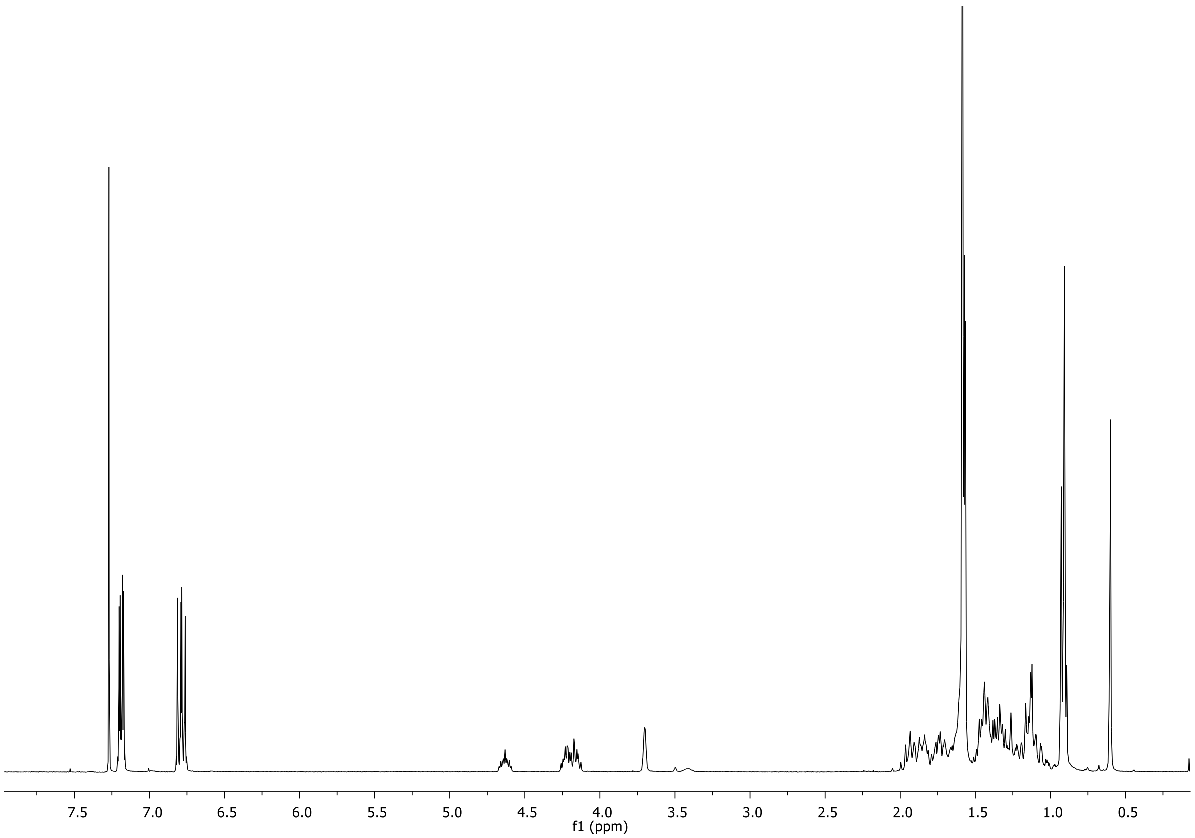

Figure S9. ^{13}C NMR of compound **3** (100 MHz, $CDCl_3$)

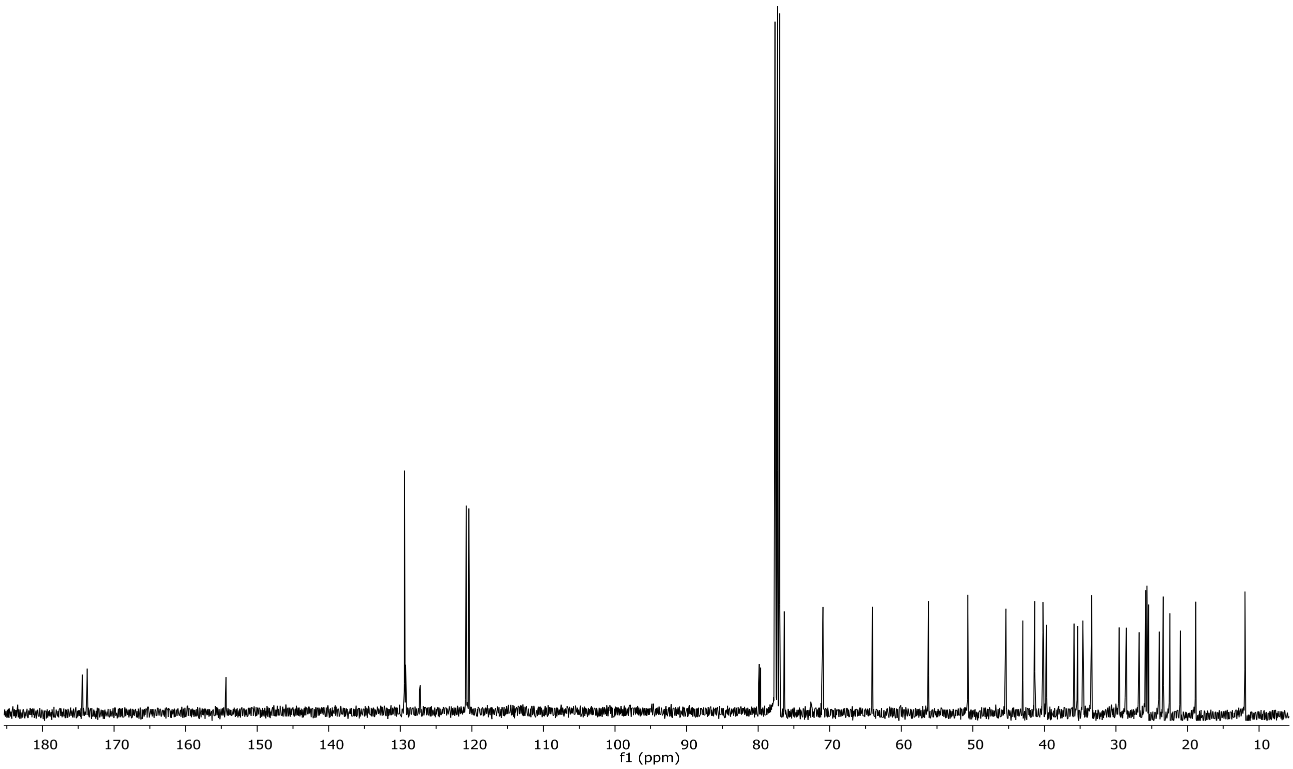

Figure S10. ^{1}H NMR of compound **4** (500 MHz, $CDCl_3$)

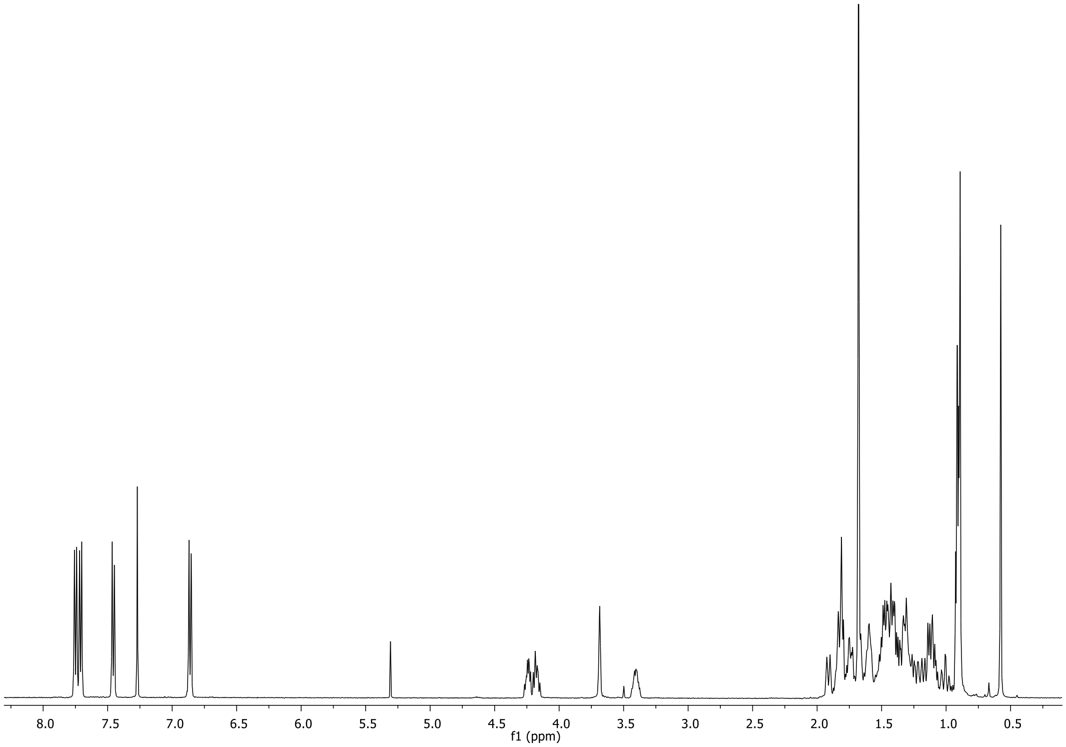

Figure S11. ^{13}C NMR of compound **4** (125 MHz, $CDCl_3$)

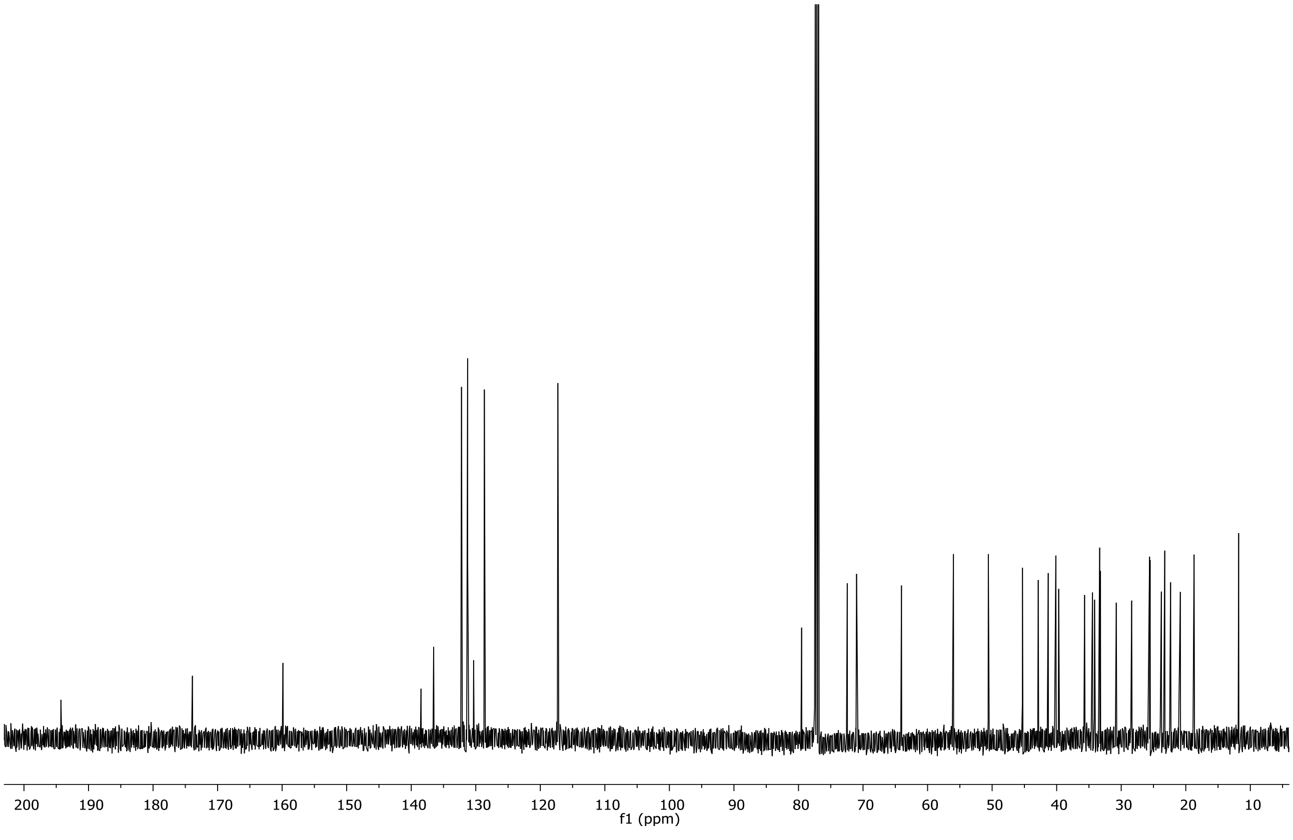

Figure S12. ^{1}H NMR of compound **5** (400 MHz, $CDCl_3$)

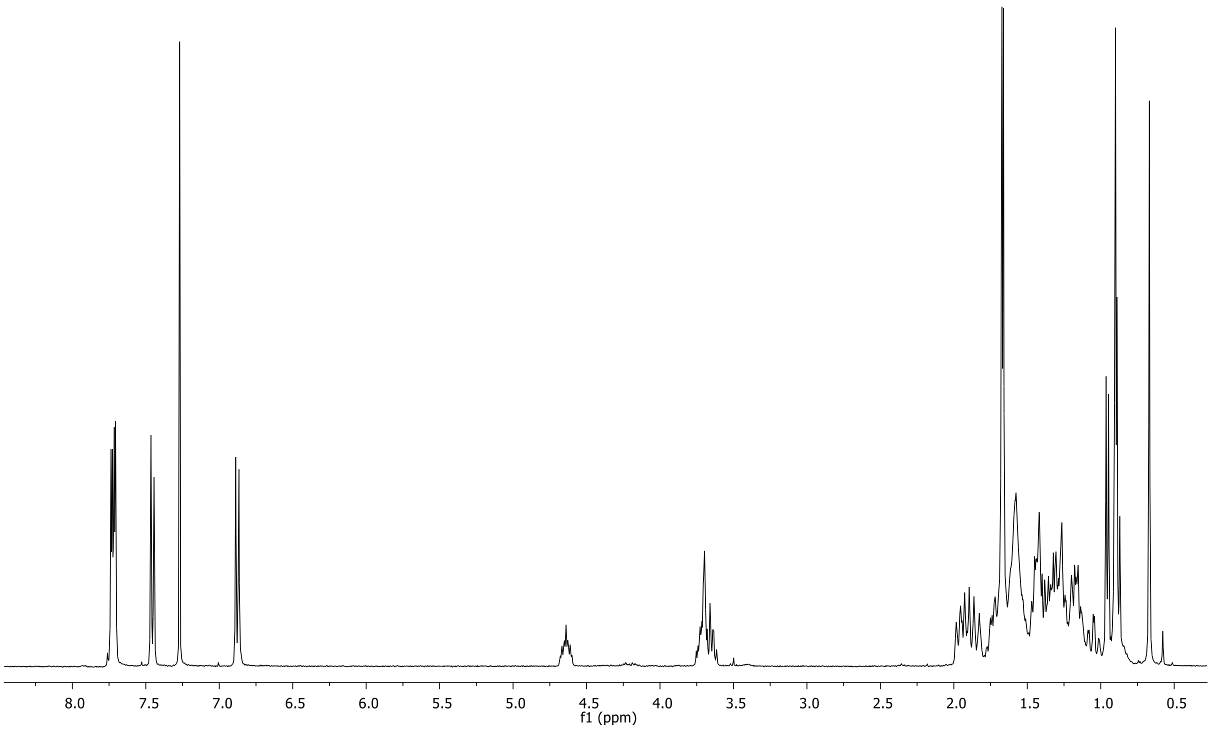

Figure S13. ^{13}C NMR of compound **5** (100 MHz, $CDCl_3$)

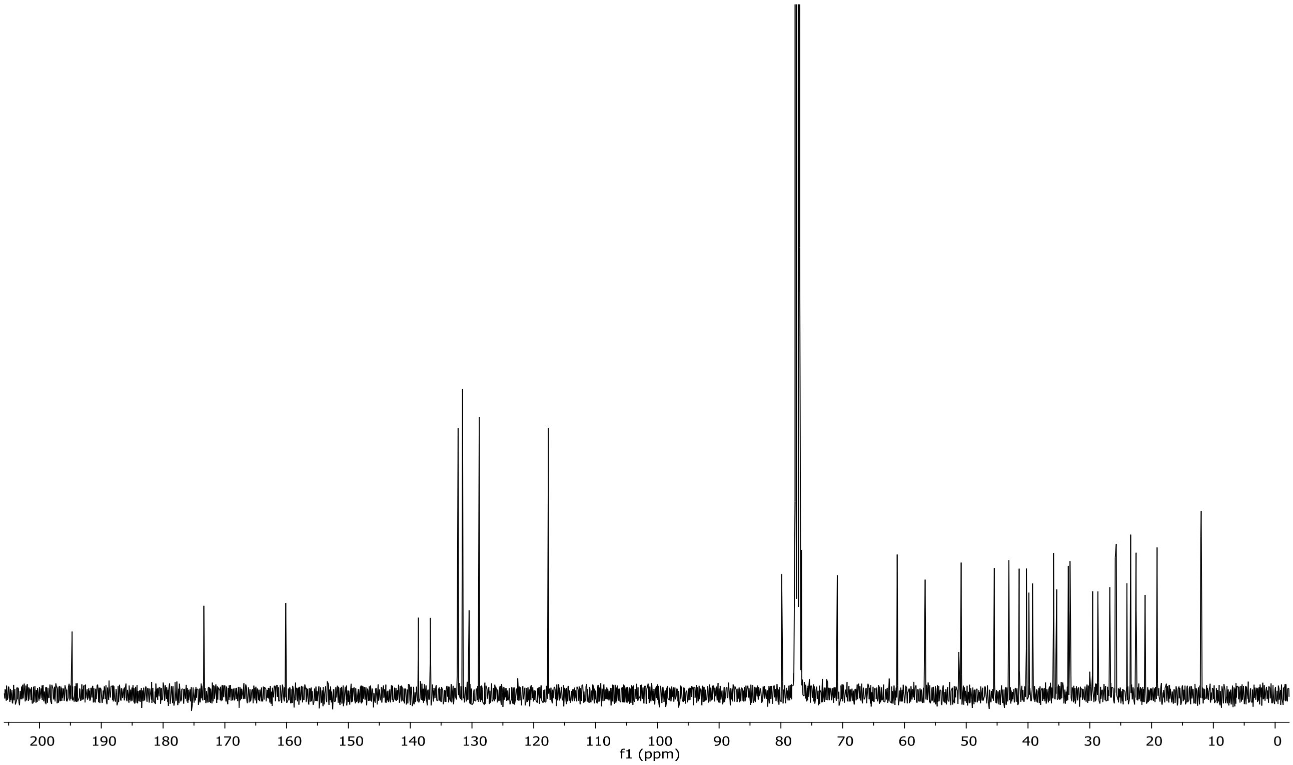

Figure S14. ^{1}H NMR of compound **6** (400 MHz, $CDCl_3$)

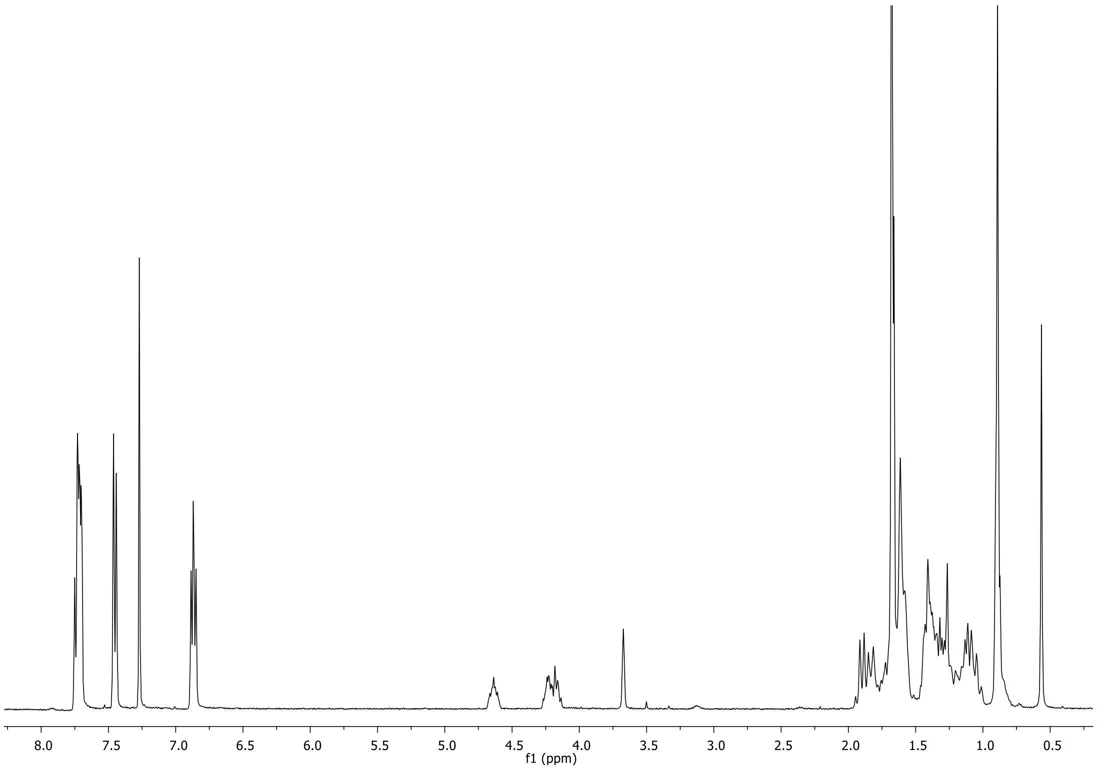

Figure S15. ^{13}C NMR of compound **6** (100 MHz, $CDCl_3$)

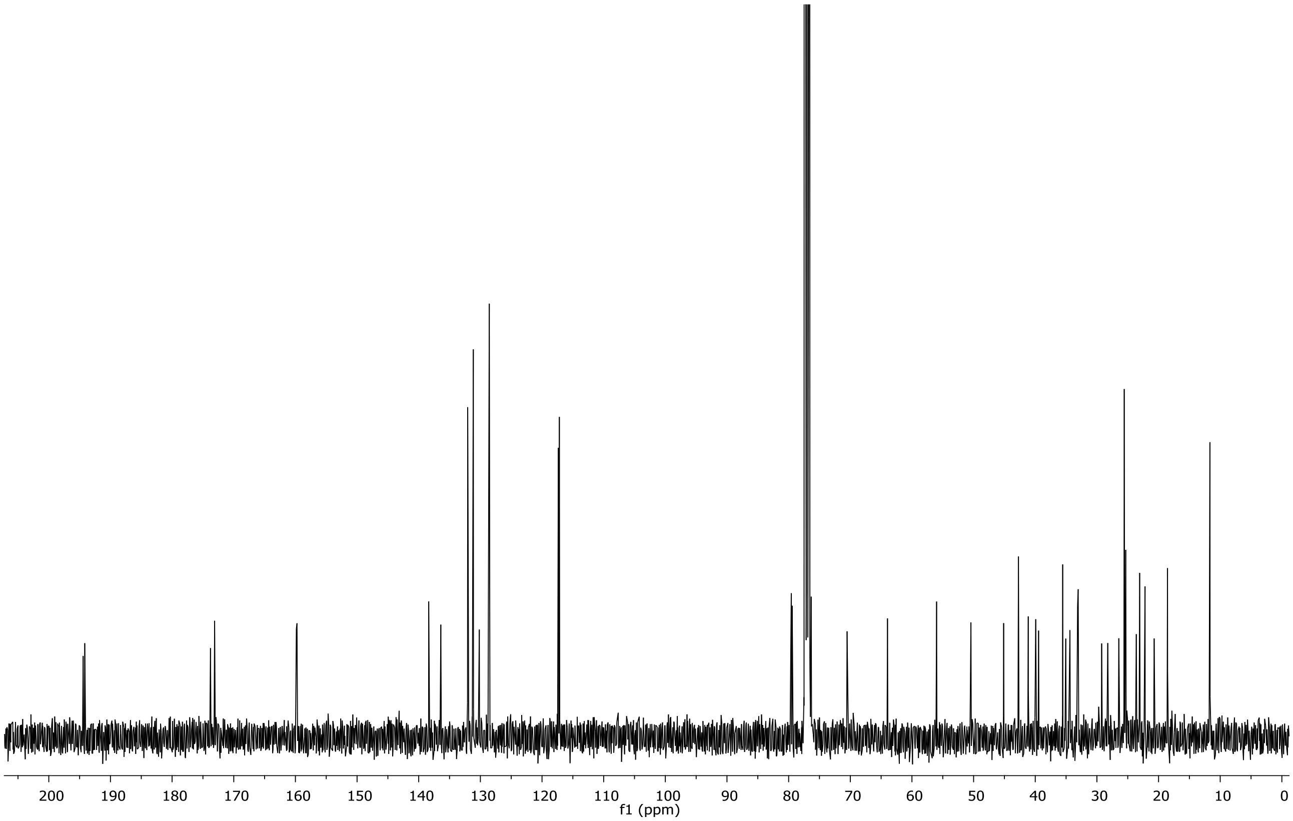

Figure S16. ^{1}H NMR of compound **7** (400 MHz, $CDCl_3$)

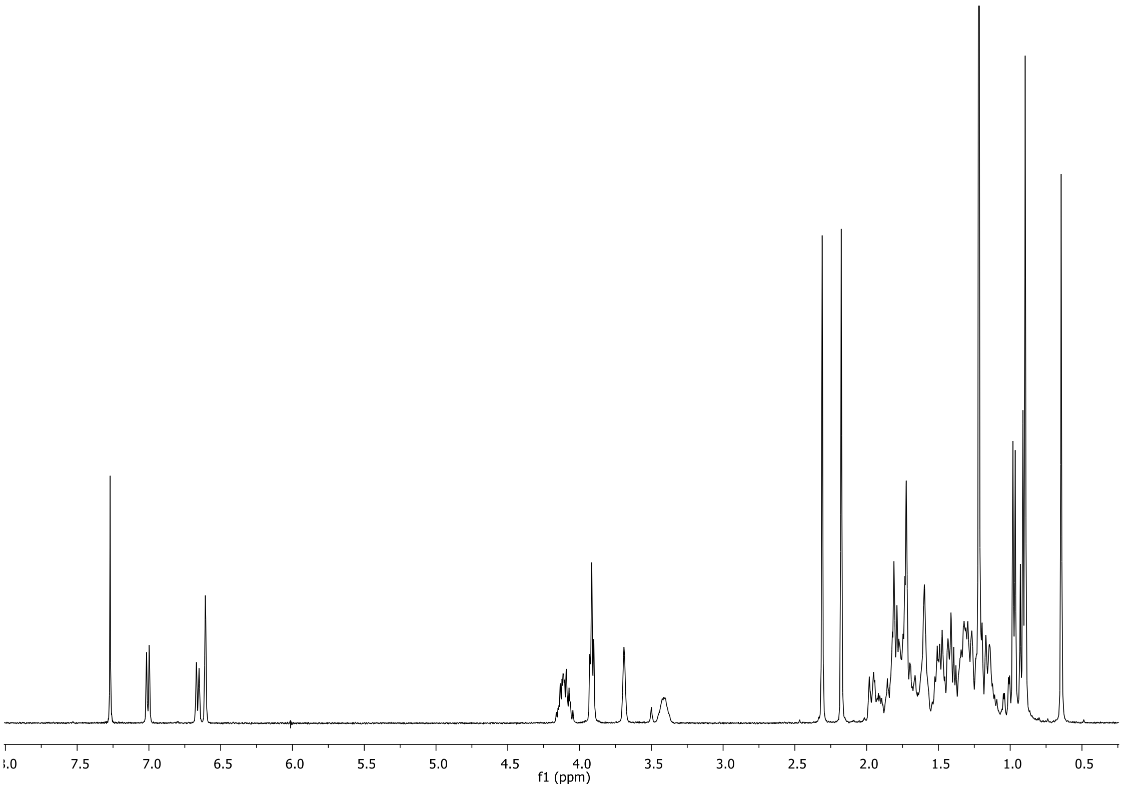

Figure S17. ^{13}C NMR of compound **7** (100 MHz, $CDCl_3$)

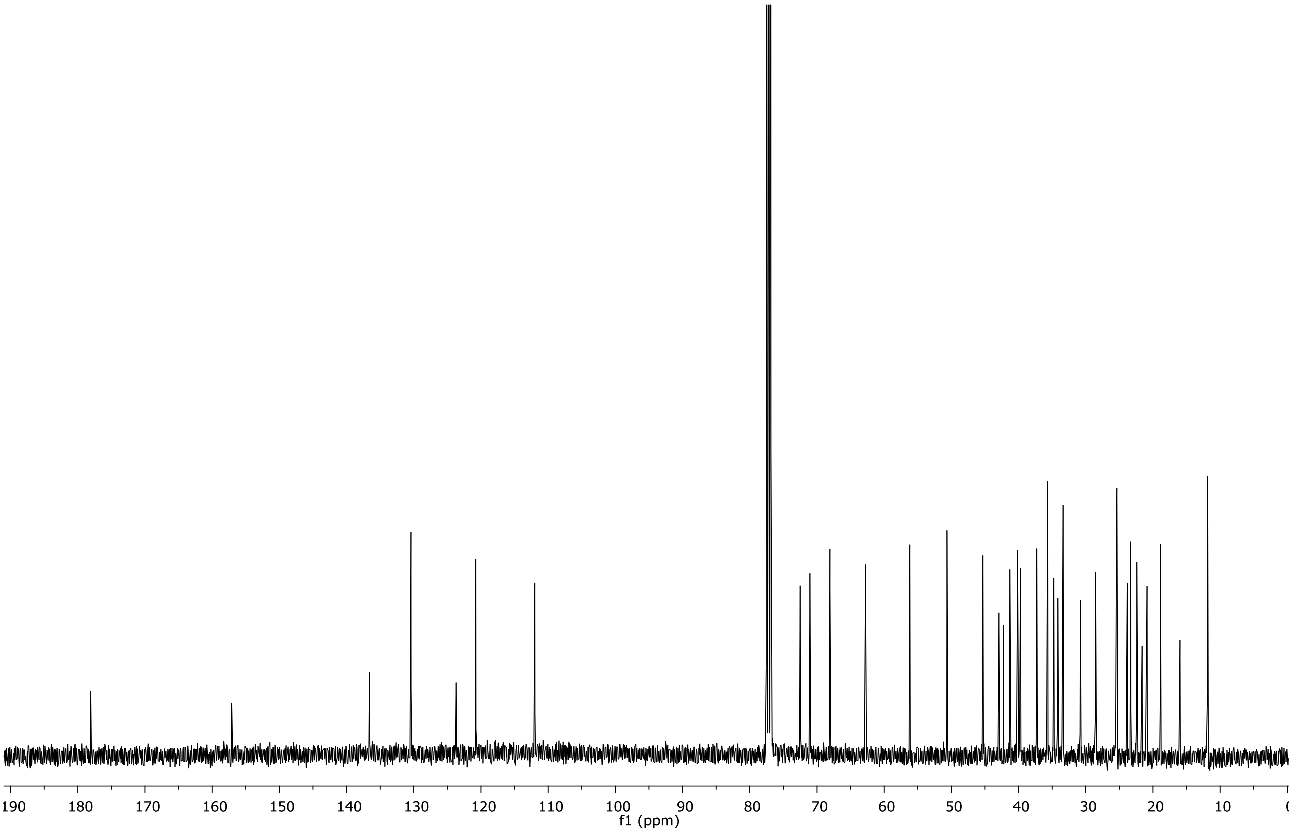

Figure S18. ^{1}H NMR of compound **8** (400 MHz, $CDCl_3$)

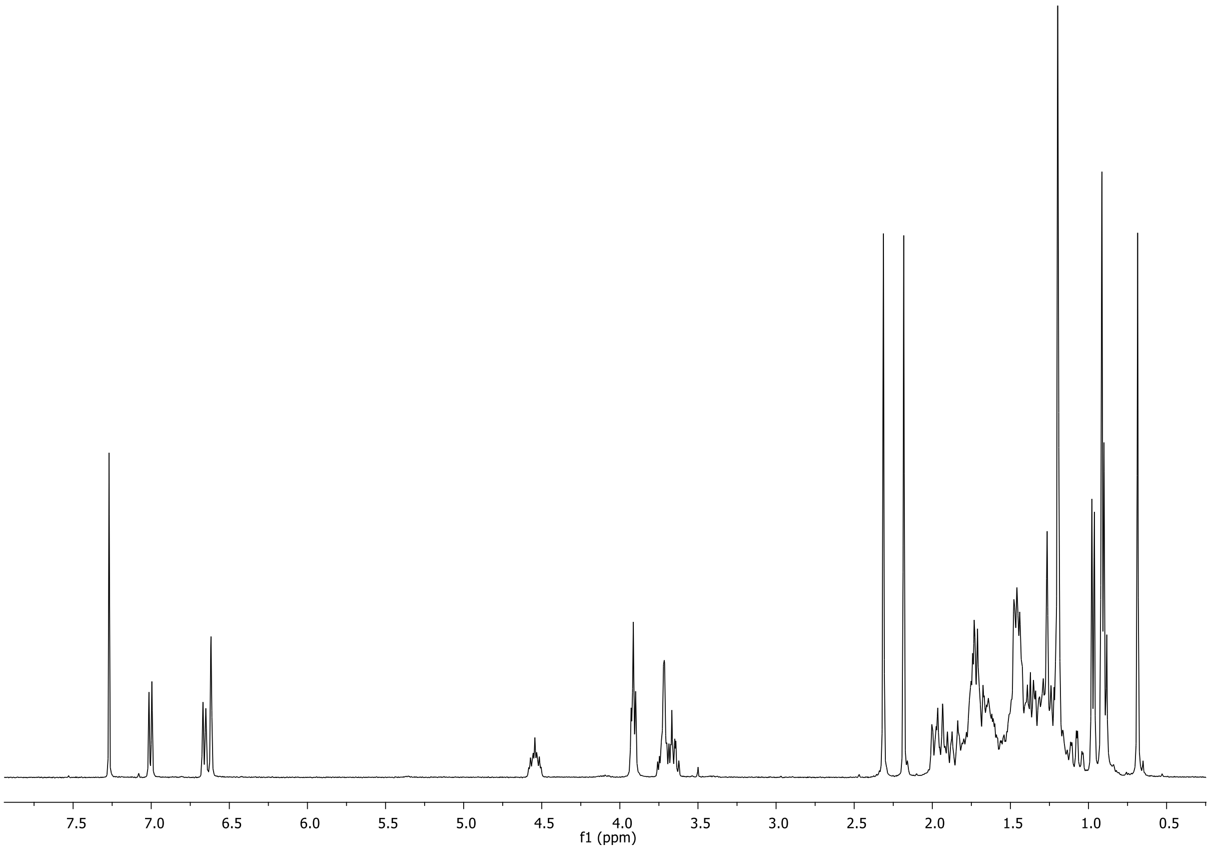

Figure S19. ^{13}C NMR of compound **8** (100 MHz, $CDCl_3$)

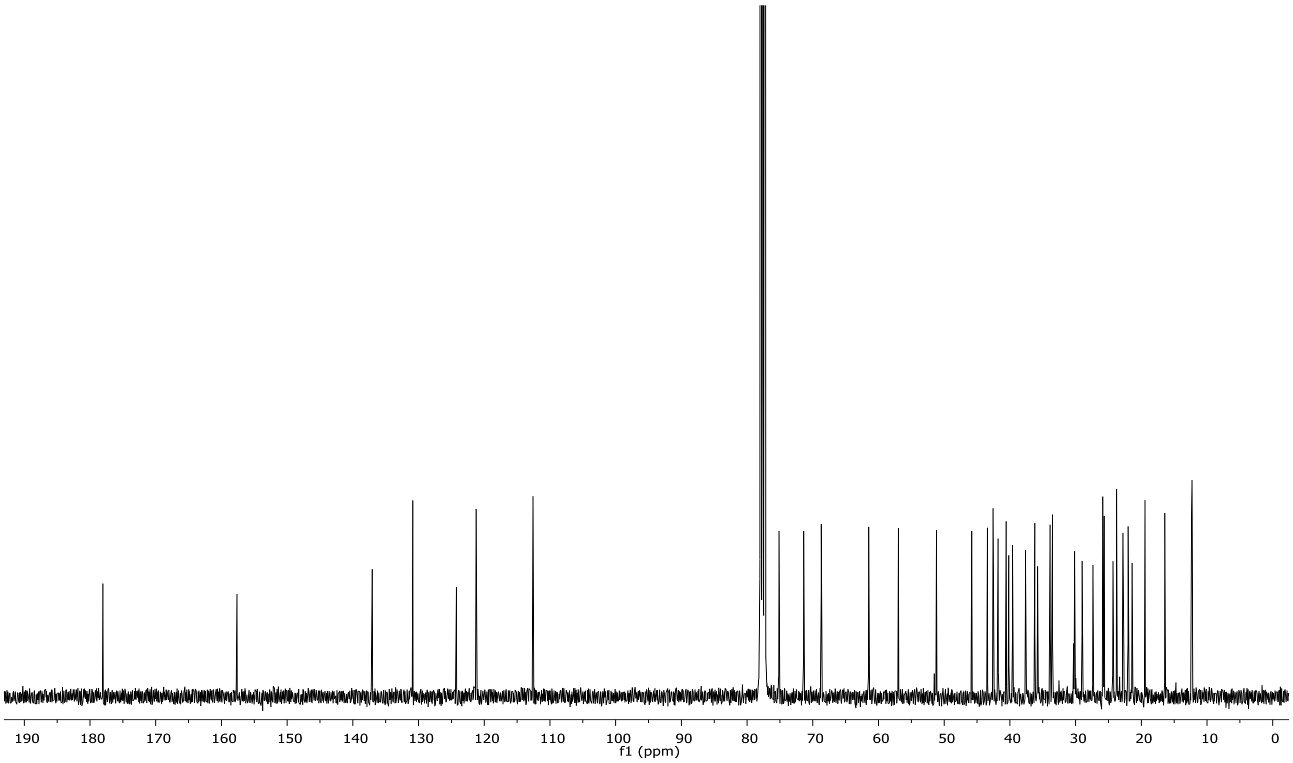

Figure S20. ^{1}H NMR of compound **9** (400 MHz, $CDCl_3$)

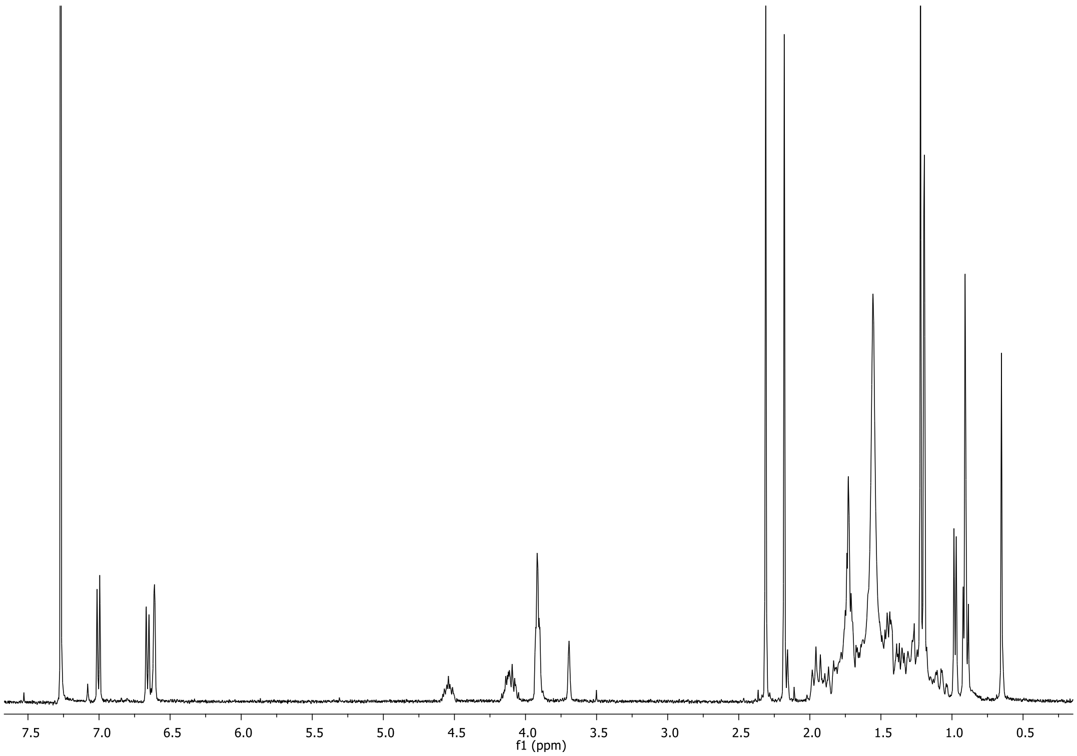

Figure S21. ^{13}C NMR of compound **9** (100 MHz, $CDCl_3$)

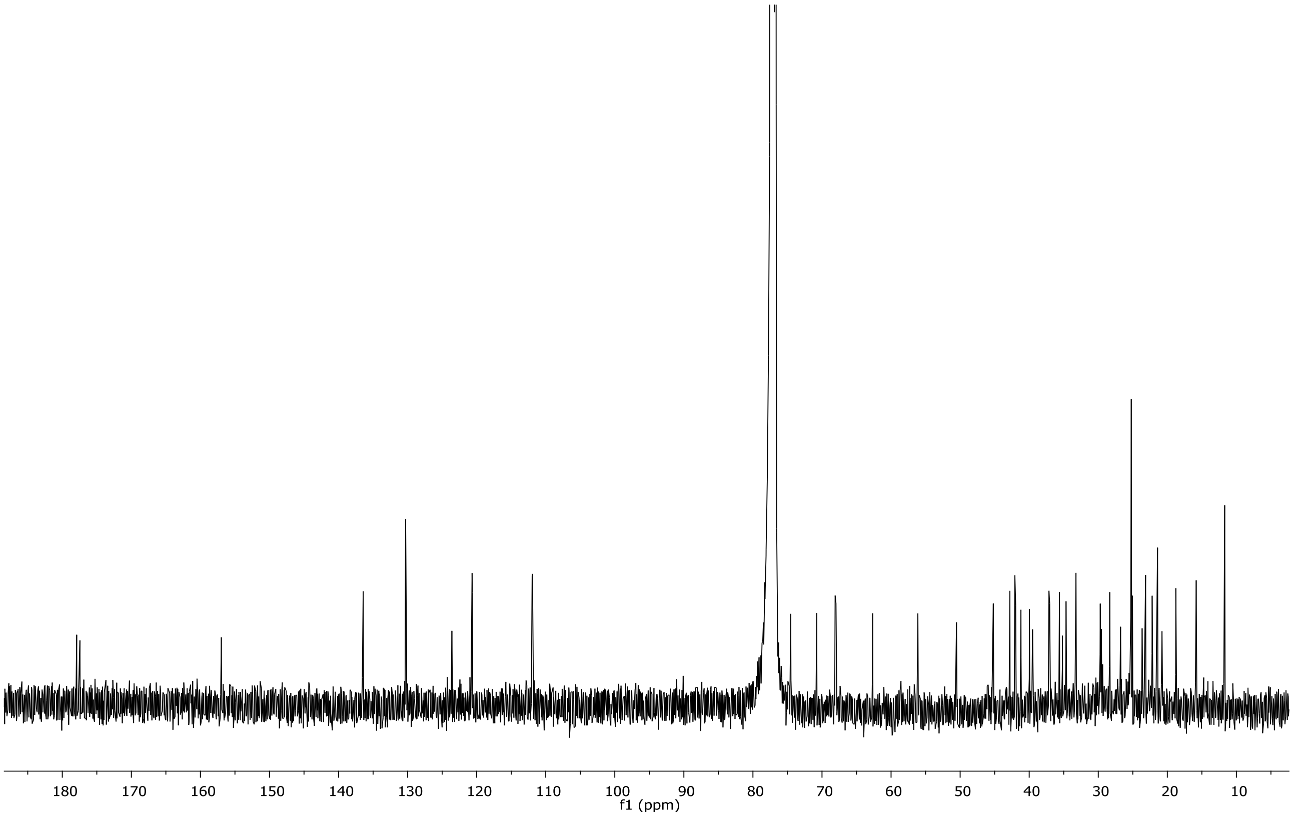

Supplement: Supplementary file 1 [file DataSheet1.PDF]
